# Supplementary material for: Single cell derived mRNA signals across human kidney tumors
Source: Nat Commun. 2021 Jun 23;12:3896. doi: 10.1038/s41467-021-23949-5 (PMC8222373; doi:10.1038/s41467-021-23949-5)
Supplement: Supplementary file 1 — Supplementary Information [file 41467_2021_23949_MOESM1_ESM.pdf]

Supplementary Figures

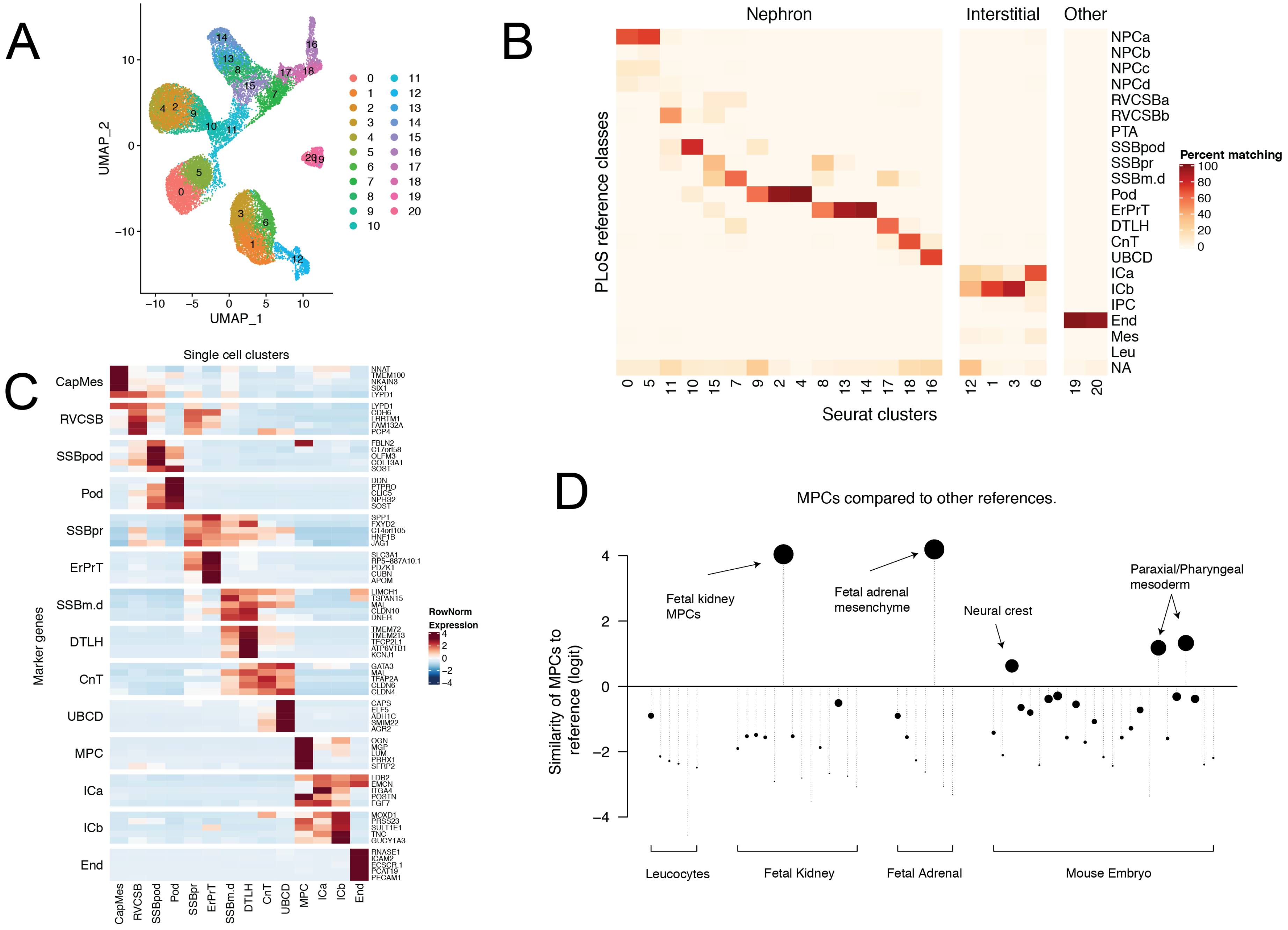

A
B
C
D
UMAP_1
UMAP_2
Nephron
Interstitial
Other
PLoS reference classes
Seurat clusters
Percent matching
Single cell clusters
Marker genes
RowNorm Expression
MPCs compared to other references.
Fetal kidney MPCs
Fetal adrenal mesenchyme
Neural crest
Paraxial/Pharyngeal mesoderm
Similarity of MPCs to reference (logit)
Leucocytes
Fetal Kidney
Fetal Adrenal
Mouse Embryo

Supplementary Figure 1 – Fetal kidney reference map

A. **UMAP of fetal kidney clusters –** Reduced dimension representation (UMAP) of the transcriptomes of 21,834 fetal kidney cells where each point represents a cell and nearby cells have similar transcriptomes. Each cell is labelled by a color corresponding to the cluster to which it belongs, as indicated by the legend on the right. Additionally, a label for each cluster is placed at the mean position of cells belonging to each cluster. Clusters are generated as discussed in the methods section.
B. **Comparison of clusters to PLoS annotation –** Each cell is assigned an annotation from[1] based on logistic regression, or NA if the similarity score is less than 1 (logit) or multiple populations have similarity greater than 1. The x-axis shows the clusters from **A,** the y-axis the reference populations and the color scale the fraction of cells assigned to each reference class. Note the high proportion of NAs in cluster 12.
C. **Marker genes defining cell types** – For each annotated cell population in **Fig. 1B**, the top 5 algorithmically determined marker genes are shown. The color scheme indicates the average normalized expression of the cells in the cluster indicated on the x-axis, z-scaled across all cell types to have mean 0 and standard deviation 1.
D. **Similarity of MPCs to other tissues** – Similarity score (logits) calculated by logistic regression trained on the fetal kidney reference to MPCs. Note that the extremely high similarity to the equivalent population in the fetal adrenal.

Source data are available as a Source Data file.

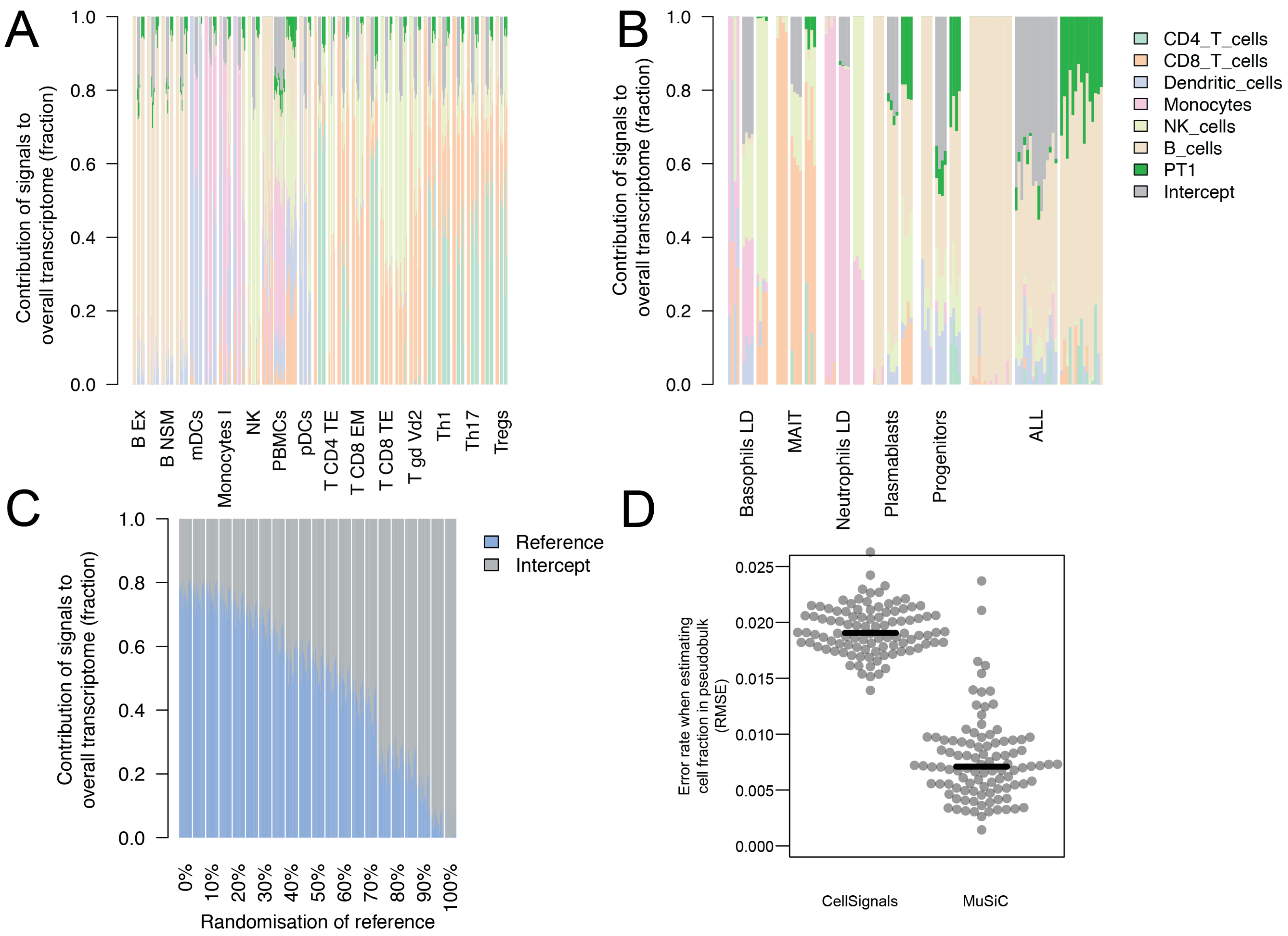

A
B
C
D
Contribution of signals to overall transcriptome (fraction)
1.0
0.8
0.6
0.4
0.2
0.0
B Ex
B NSM
mDCs
Monocytes I
NK
PBMCs
pDCs
T CD4 TE
T CD8 EM
T CD8 TE
T gd Vd2
Th1
Th17
Tregs
Basophils LD
MAIT
Neutrophils LD
Plasmablasts
Progenitors
ALL
CD4_T_cells
CD8_T_cells
Dendritic_cells
Monocytes
NK_cells
B_cells
PT1
Intercept
Reference
Intercept
0%
10%
20%
30%
40%
50%
60%
70%
80%
90%
100%
Randomisation of reference
Error rate when estimating cell fraction in pseudobulk (RMSE)
0.025
0.020
0.015
0.010
0.005
0.000
CellSignals
MuSiC

Supplementary Figure 2 – Benchmarking of cellular signal analysis

A. **Benchmarking using bulk transcriptomes with match in reference –** The same comparison as in **Fig. 1D** but where each reference cell signal is shown individiually rather than grouped into "matching" and "non-matching". Each bar represents a bulk transcriptome, with the different colors indicating the relative contribution from each reference cell signal (see legend). Transcriptomes are grouped into blocks of the same type as shown on the x-axis and within each block the results of deconvolution using BSeq-SC, cellular signal analysis, and MuSiC are shown in separate blocks from left to right.
B. **Benchmarking using bulk transcriptomes with no match in reference –** The same comparison as in **Fig. 1E** but where each reference cell signal is shown individiually rather than grouped into "matching" and "non-matching". Each bar represents a bulk transcriptome, with the different colors indicating the relative contribution from each reference cell signal (see legend). Transcriptomes are grouped into blocks of the same type as shown on the x-axis and within each block the results of deconvolution using BSeq-SC, cellular signal analysis, and MuSiC are shown in separate blocks from left to right.
C. **Response of unexplained signal (Intercept) to incomplete reference** – Bulk transcriptomes from flow sorted B cells are fit using cellular signal analysis using a reference consisting of a B cell signal only. This reference is then progressively randomized to decrease its suitability for the bulk B cell transcriptomes as indicated by the label on the x-axis. In each case, the resulting fraction of the signal that is allocated to either the modified reference, or the intercept term (the "unexplained signal") is shown by the size of the colored bars.
D. **Recovering composition of bulk tissue from bulk transcriptomes** – Using the same single cell PBMC data used as a reference in panels A and B, 100 pseudobulk transcriptomes were generated by random sampling. These pseudobulk transcriptomes were then deconvoluted with both cell signal analysis and MuSiC using the same single cell data as a reference. Finally, the abundance of cell types in each pseudobulk transcriptome was estimated and compared to the true proportions to calculate the root mean squared error (RMSE) for each sample. The RMSE values for all 100 simulations are shown on the y-axis, and the deconvolution method is shown on the x-axis. The black line indicates the median RMSE for each method.

Source data are available as a Source Data file.

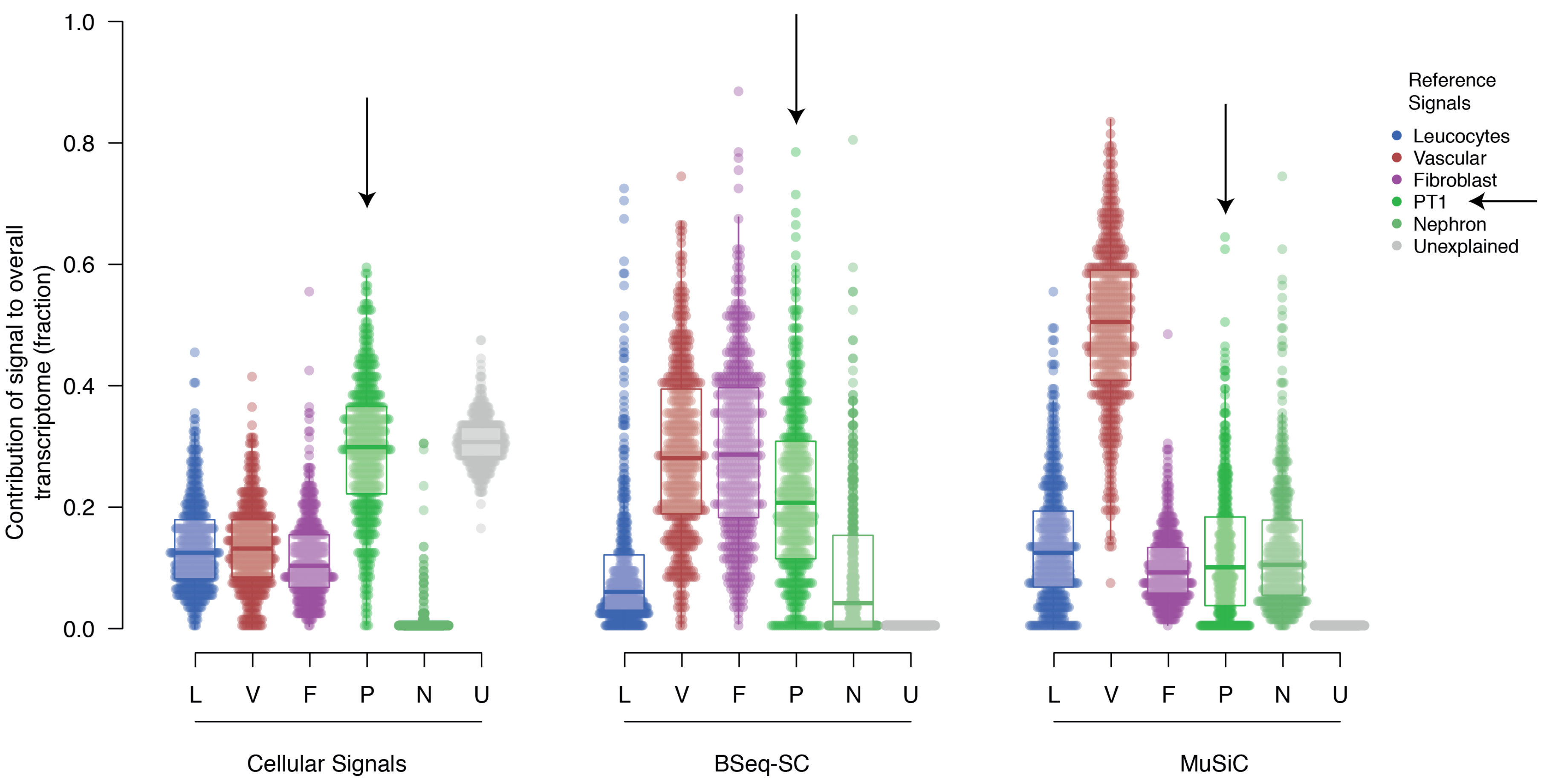

1.0
0.8
0.6
0.4
0.2
0.0
Contribution of signal to overall transcriptome (fraction)
L V F P N U
Cellular Signals
L V F P N U
BSeq-SC
L V F P N U
MuSiC
Reference Signals
Leucocytes
Vascular
Fibroblast
PT1
Nephron
Unexplained

Supplementary Figure 3 – Ability of different methods to recover known RCC signal

We applied our cellular signal method, BSEQ-sc and MuSiC to a population of clear cell and papillary cell renal cell carcinomas, using reference signals derived from the mature normal kidney. This population of tumors is known to resemble the PT1 population of proximal tubular cells and have a strong vascular component. The relative contribution of each signal to each bulk RNA-seq sample is shown by the y-axis. The results for the three methods are split into blocks, with cellular signals on the left, BSEQ-sc in the middle, and MuSiC on the right. Each signal type is labelled with an abbreviation and colored as shown by the legend on the right. Signals are marked with a square for fetal kidney and circle for mature kidney. For our method, there is an additional signal which represents the unexplained signal for each sample (see Methods). Contributions due to vascular signals, fibroblast signals, leucocyte signals, and all non-proximal tubular nephron signals are aggregated together. Each signal/sample combination is represented by a single point and the distribution of relative signal contributions to the bulk transcriptomes are summarized with boxplots. Arrows mark the PT1 population which these tumors are known to resemble. Boxplots show the distribution median (middle line), 1st and 3rd quartiles (box limits), and 1.5 times the inter-quartile range beyond the box-limits (whiskers).

Source data are available as a Source Data file.

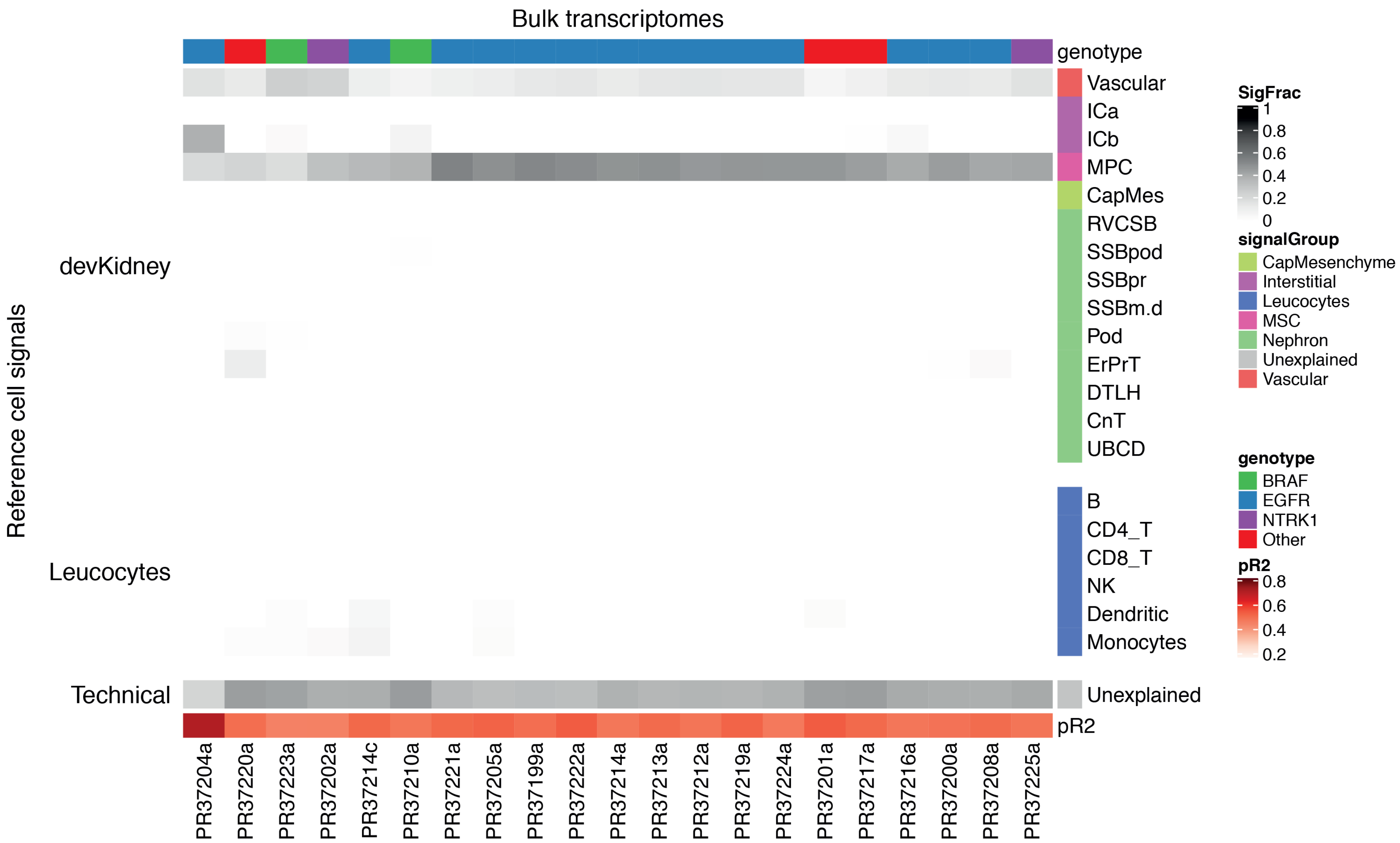
Bulk transcriptomes
genotype
Vascular
ICa
ICb
MPC
CapMes
RVCSB
SSBpod
SSBpr
SSBm.d
Pod
ErPrT
DTLH
CnT
UBCD
B
CD4_T
CD8_T
NK
Dendritic
Monocytes
Unexplained
pR2
Reference cell signals
devKidney
Leucocytes
Technical
PR37204a
PR37220a
PR37223a
PR37202a
PR37214c
PR37210a
PR37221a
PR37205a
PR37199a
PR37222a
PR37214a
PR37213a
PR37212a
PR37219a
PR37224a
PR37201a
PR37217a
PR37216a
PR37200a
PR37208a
PR37225a
SigFrac
1
0.8
0.6
0.4
0.2
0
signalGroup
CapMesenchyme
Interstitial
Leucocytes
MSC
Nephron
Unexplained
Vascular
genotype
BRAF
EGFR
NTRK1
Other
pR2
0.8
0.6
0.4
0.2

Supplementary Figure 4 – CMNs resembles interstitial cells

The same data as in **Fig. 3A**, but presented in heatmap form. Each column represents a sample and each row a collection of reference signal. The shading in each cell represents the relative contribution of that collection of reference signals to explaining the bulk transcriptome of that sample. The CMN samples are then further sub-divided by genotype. Sample IDs are printed below each column. The pR2 column represents a pseudo-R squared value for each sample, calculated as 1 minus the ratio of the log likelihoods of the full model to a model consisting of only the intercept term.

Source data are available as a Source Data file.

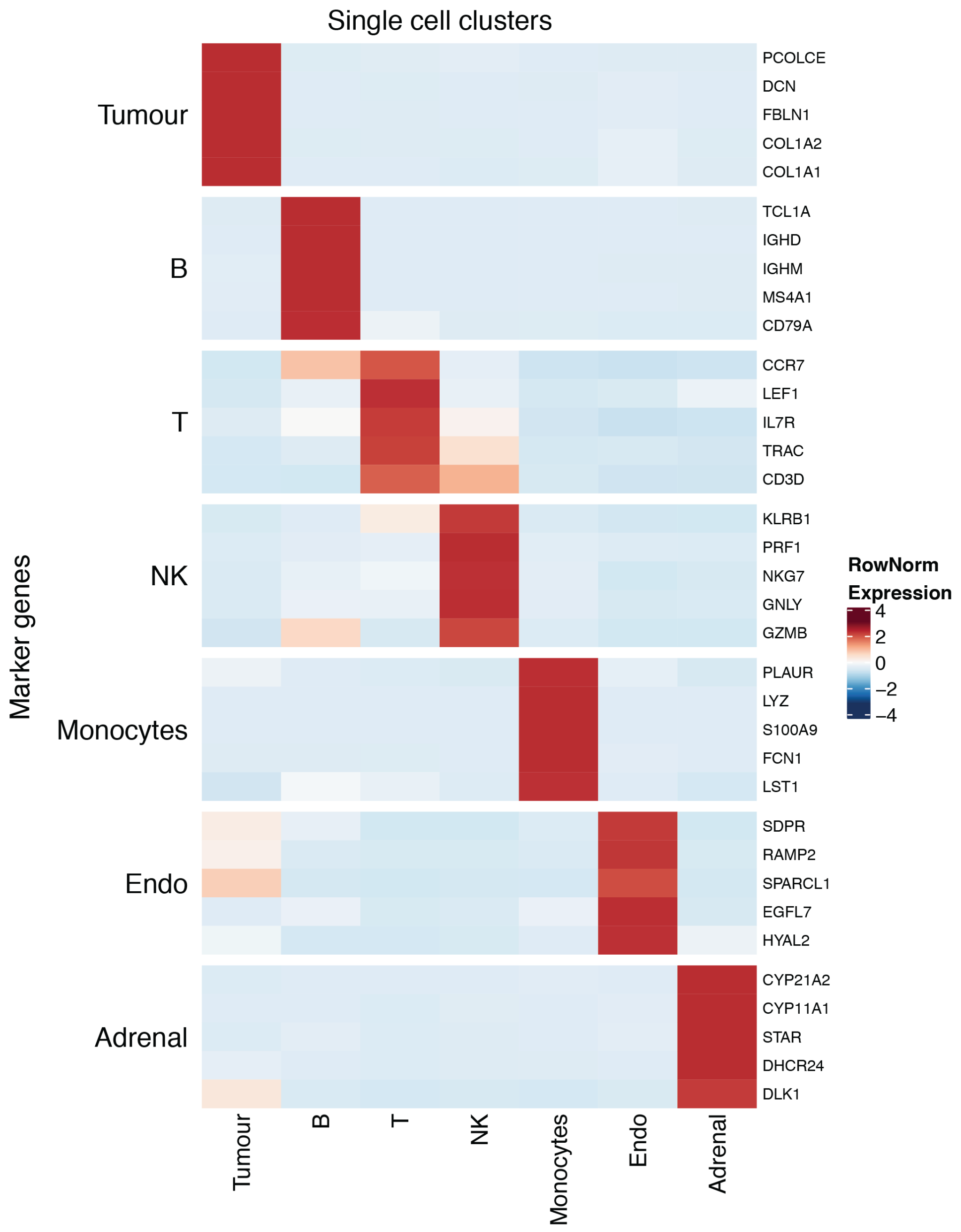

Supplementary Figure 5 – Key markers of cell types of CMN For each annotated cell population in **Fig. 3D**, the top 5 algorithmically determined marker genes are shown. The color scheme indicates the average normalized expression of the cells in the cluster indicated on the x-axis, z-scaled across all cell types to have mean 0 and standard deviation 1. Source data are available as a Source Data file.

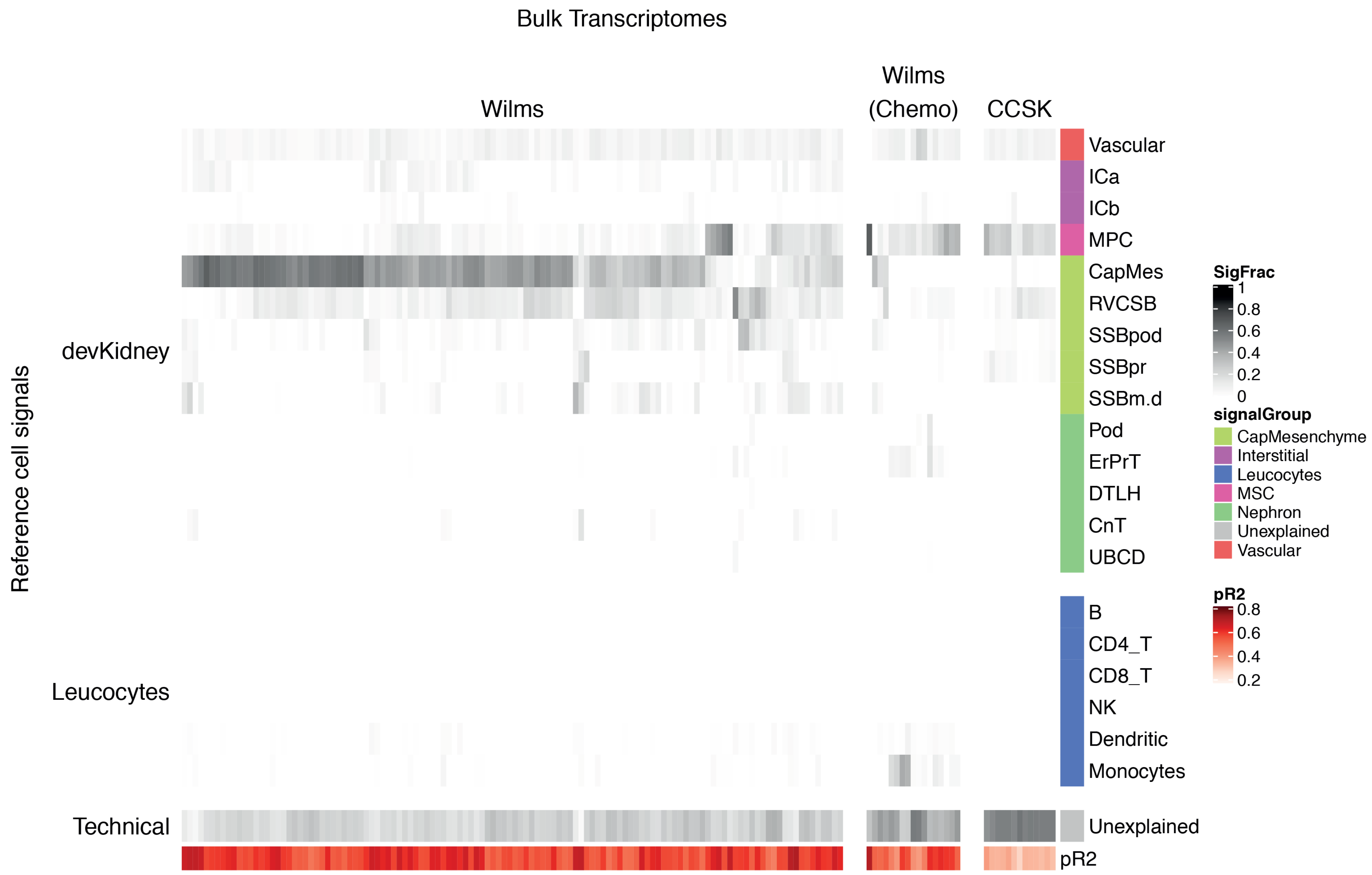
Bulk Transcriptomes
Wilms
Wilms (Chemo)
CCSK
Reference cell signals
devKidney
Leucocytes
Technical
Vascular
ICa
ICb
MPC
CapMes
RVCSB
SSBpod
SSBpr
SSBm.d
Pod
ErPrT
DTLH
CnT
UBCD
B
CD4_T
CD8_T
NK
Dendritic
Monocytes
Unexplained
pR2
SigFrac
1
0.8
0.6
0.4
0.2
0
signalGroup
CapMesenchyme
Interstitial
Leucocytes
MSC
Nephron
Unexplained
Vascular
pR2
0.8
0.6
0.4
0.2

Supplementary Figure 6 – Wilms/Nephroblastoma and CCSK fit using fetal kidney signals
The same data as in **Fig. 4A**, but presented in heatmap form. Each column represents a sample and each row a collection of reference signal. The shading in each cell represents the relative contribution of that collection of reference signals to explaining the bulk transcriptome of that sample. The pR2 column represents a pseudo-R squared value for each sample, calculated as 1 minus the ratio of the log likelihoods of the full model to a model consisting of only the intercept term.
Source data are available as a Source Data file.

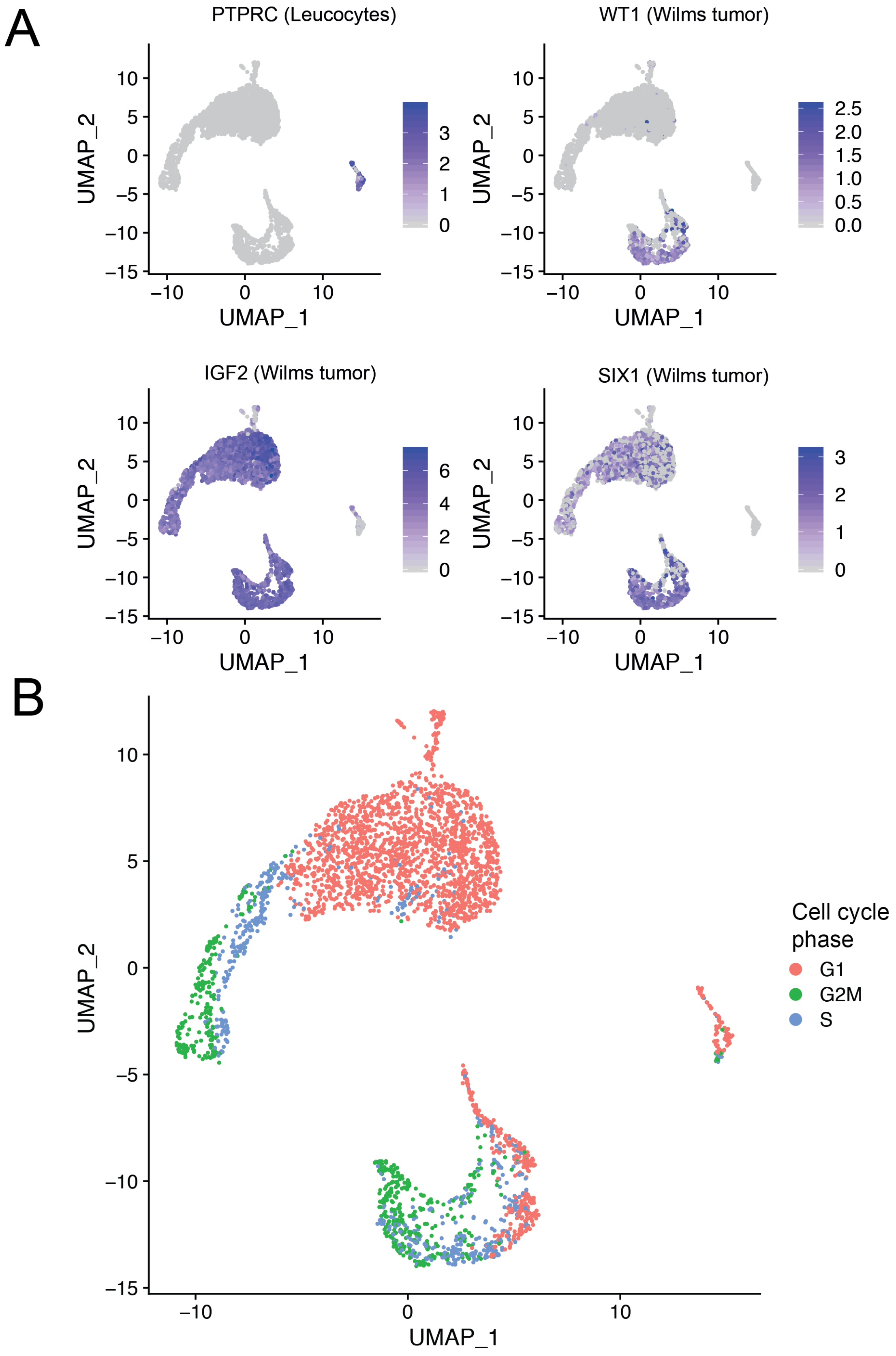

A
PTPRC (Leucocytes)
WT1 (Wilms tumor)
IGF2 (Wilms tumor)
SIX1 (Wilms tumor)
UMAP_1
UMAP_2
B
Cell cycle phase
G1
G2M
S

Supplementary Figure 7 – Annotation of single cell Wilms data

A. **Expression of key markers –** Reduced dimension representation (UMAP) of the transcriptomes of Wilms cells, colored by log-normalized expression of the gene in the panel title. The cell type that a gene is a marker of is shown in brackets.
B. **Phase of cell cycle –** The same data as panel **A**, but colored by inferred cell cycle phase as indicated by the legend on the right.

Source data are available as a Source Data file.

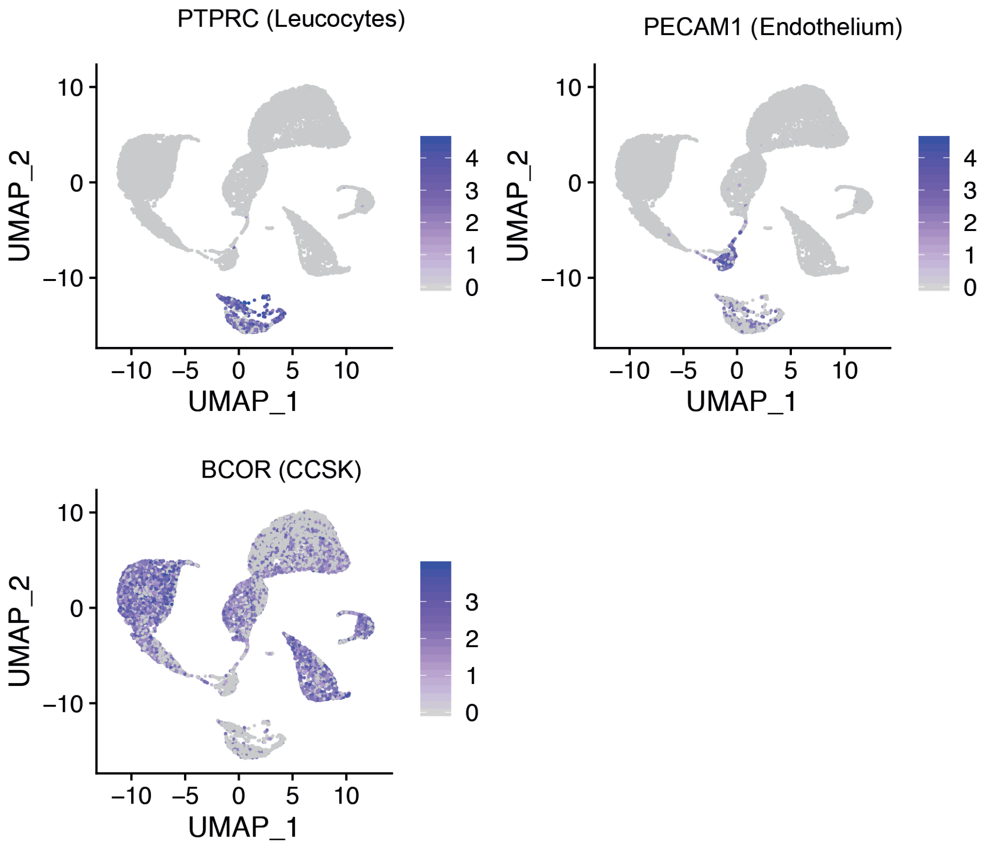

Supplementary Figure 8 – Annotation of single cell CCSK data

Reduced dimension representation (UMAP) of the transcriptomes of CCSK cells, colored by log-normalized expression of the gene in the panel title. The cell type that a gene is a marker of is shown in brackets.

Source data are available as a Source Data file.

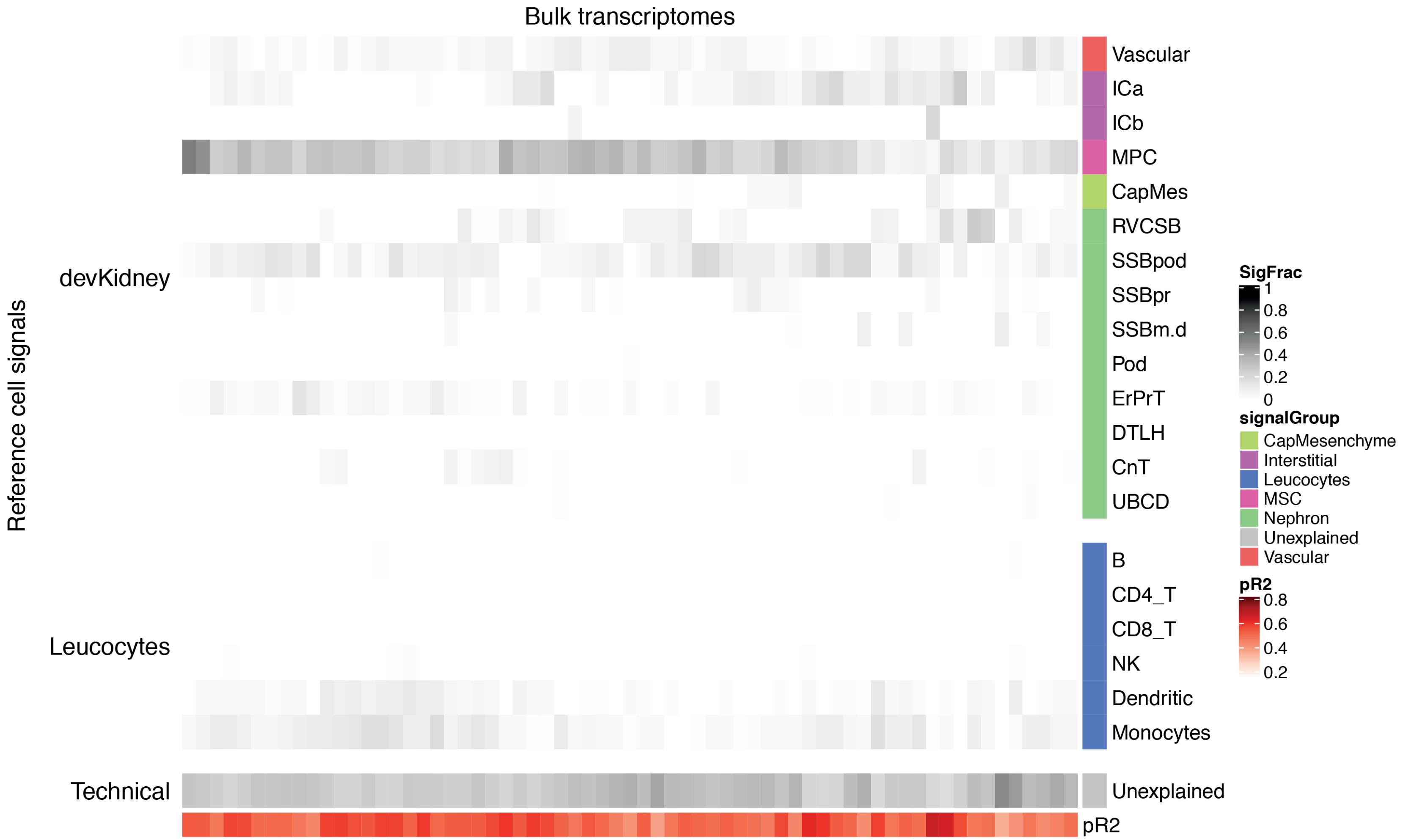

Bulk transcriptomes
Reference cell signals
devKidney
Leucocytes
Technical
Vascular
ICa
ICb
MPC
CapMes
RVCSB
SSBpod
SSBpr
SSBm.d
Pod
ErPrT
DTLH
CnT
UBCD
B
CD4_T
CD8_T
NK
Dendritic
Monocytes
Unexplained
pR2
SigFrac
1
0.8
0.6
0.4
0.2
0
signalGroup
CapMesenchyme
Interstitial
Leucocytes
MSC
Nephron
Unexplained
Vascular
pR2
0.8
0.6
0.4
0.2

Supplementary Figure 9 – MRTs fit using fetal kidney signals

The same data as in **Fig. 5A**, but presented in heatmap form. Each column represents a sample and each row a collection of reference signal. The shading in each cell represents the relative contribution of that collection of reference signals to explaining the bulk transcriptome of that sample. The pR2 column represents a pseudo-R squared value for each sample, calculated as 1 minus the ratio of the log likelihoods of the full model to a model consisting of only the intercept term.

Source data are available as a Source Data file.

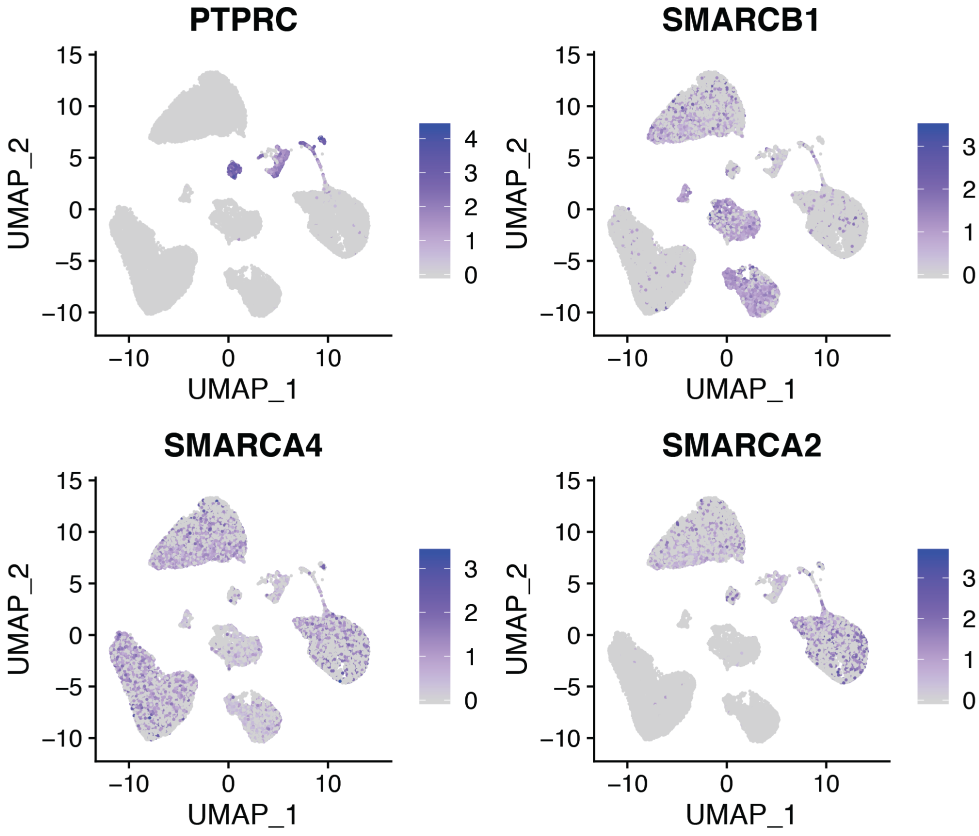

Supplementary Figure 10 - Annotation of single cell MRT data

Reduced dimension representation (UMAP) of the transcriptomes of MRT cells, colored by log-normalized expression of the gene in the panel title.

Source data are available as a Source Data file.

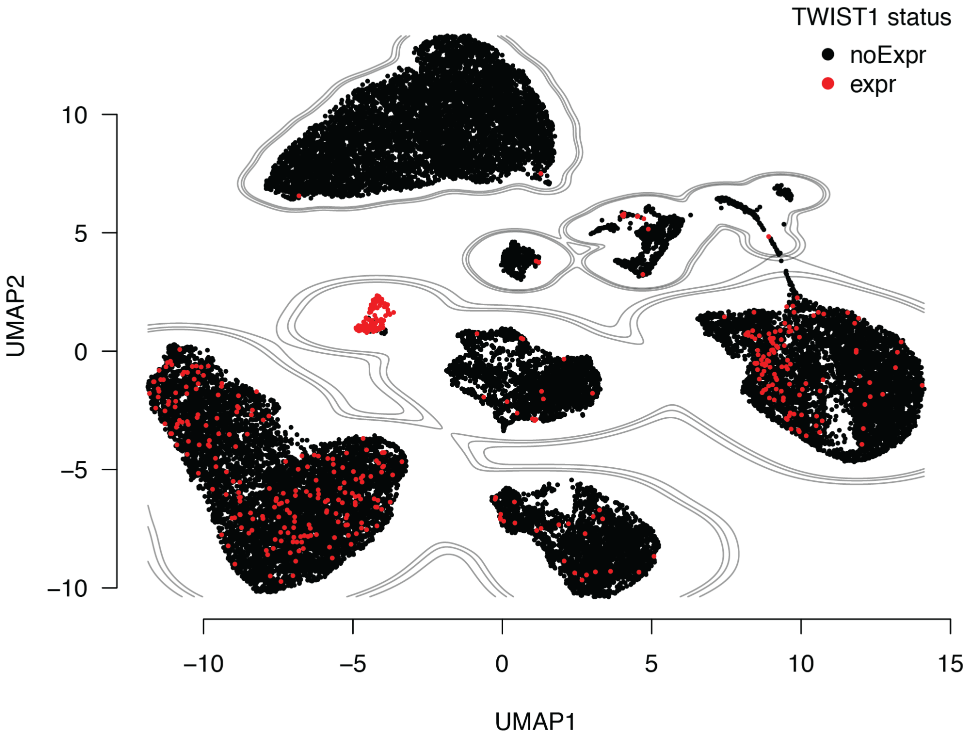

Supplementary Figure 11 – TWIST1 positive cells in MRT
Reduced dimension representation (UMAP) of the transcriptomes of MRT cells (as in **Fig. 5B**), with cells with any expression of *TWIST1* colored red and those without colored black. Source data are available as a Source Data file.

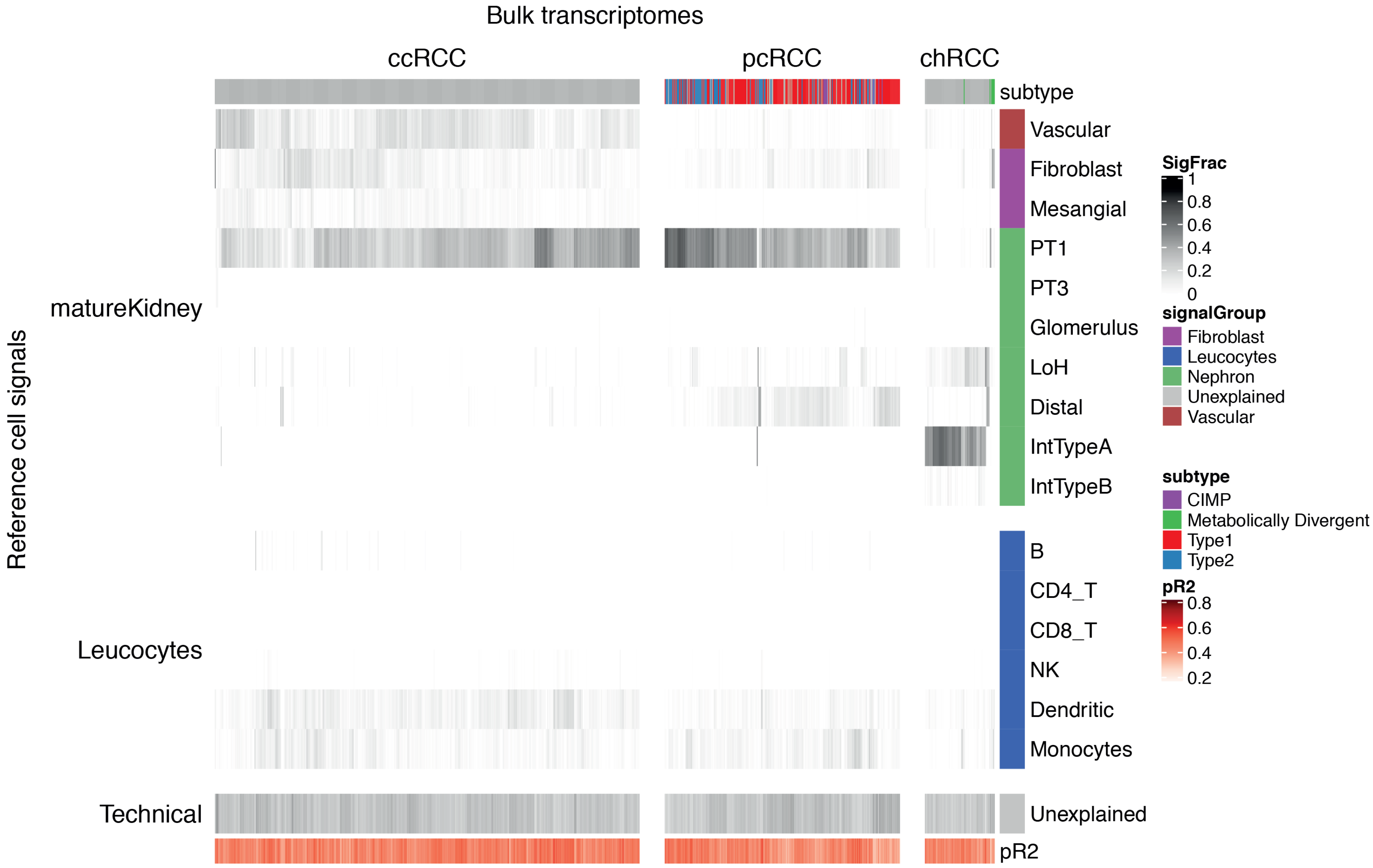

Bulk transcriptomes
ccRCC
pcRCC
chRCC
subtype
Vascular
Fibroblast
Mesangial
PT1
PT3
Glomerulus
LoH
Distal
IntTypeA
IntTypeB
B
CD4_T
CD8_T
NK
Dendritic
Monocytes
Unexplained
pR2
Reference cell signals
matureKidney
Leucocytes
Technical
SigFrac
1
0.8
0.6
0.4
0.2
0
signalGroup
Fibroblast
Leucocytes
Nephron
Unexplained
Vascular
subtype
CIMP
Metabolically Divergent
Type1
Type2
pR2
0.8
0.6
0.4
0.2

Supplementary Figure 12 – All RCCs fit using mature kidney signals

The same data as in **Fig. 6A**, but presented in heatmap form. Each column represents a sample and each row a collection of reference signal. Subtypes of tumor are indicated by the annotation row at the top of the figure. The shading in each cell represents the relative contribution of that collection of reference signals to explaining the bulk transcriptome of that sample. The pR2 column represents a pseudo-R squared value for each sample, calculated as 1 minus the ratio of the log likelihoods of the full model to a model consisting of only the intercept term. Source data are available as a Source Data file.

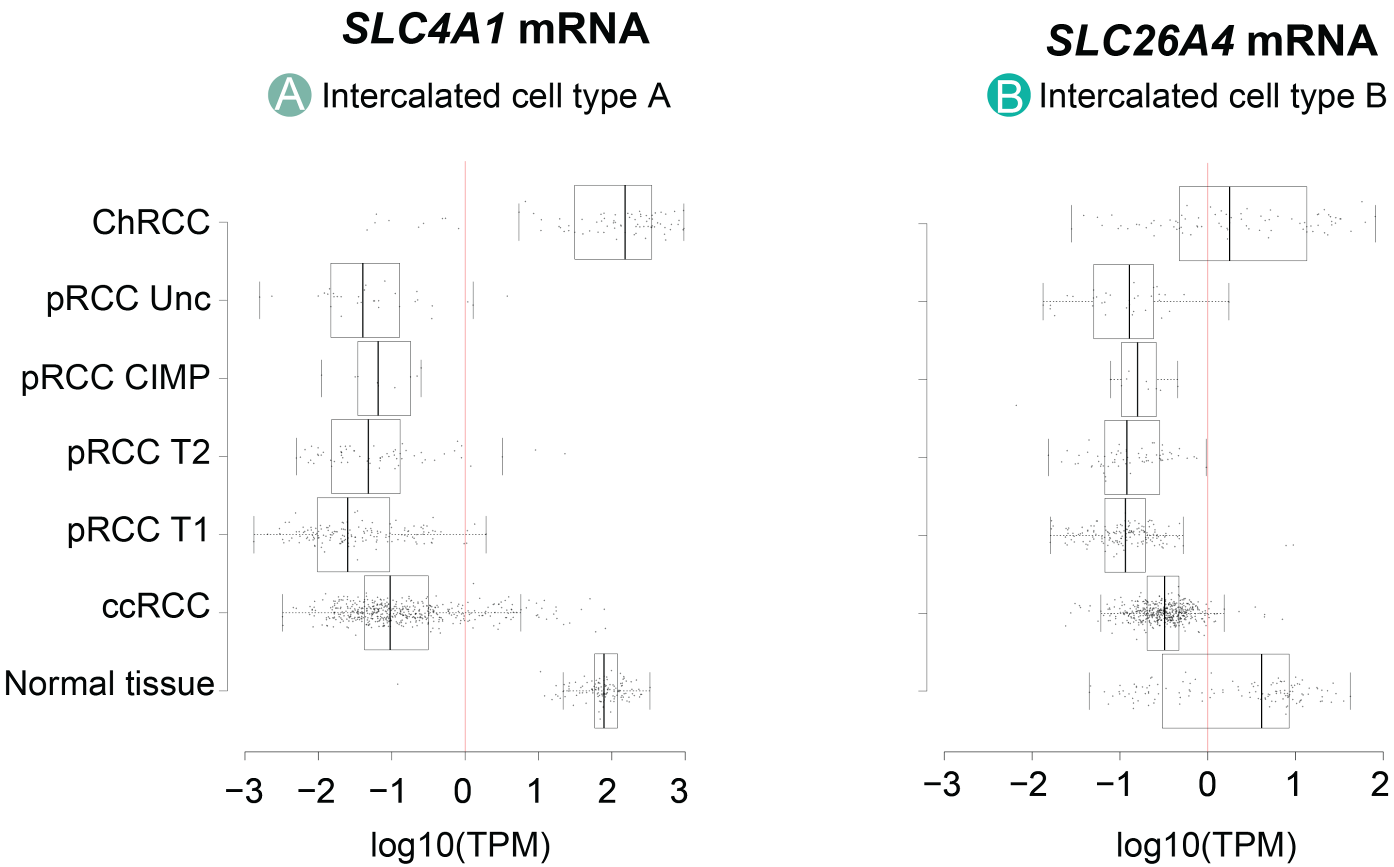

Supplementary Figure 13 - Expression of markers of Type A and B intercalated cells in bulk transcriptomes
Boxplots showing expression of canonical markers of Type A (left) and B (right) intercalated cells in different populations of bulk kidney tumor and normal transcriptomes. Expression values are normalized to transcripts per million (TPM) and log10 transformed and shown on the x-axis, with each point representing a sample and a boxplot showing the distribution of expression values for that sample type. A red line marks 0 on the log transformed expression scale. Boxplots show the distribution median (middle line), 1st and 3rd quartiles (box limits), and 1.5 times the inter-quartile range beyond the box-limits (whiskers).
Source data are available as a Source Data file.

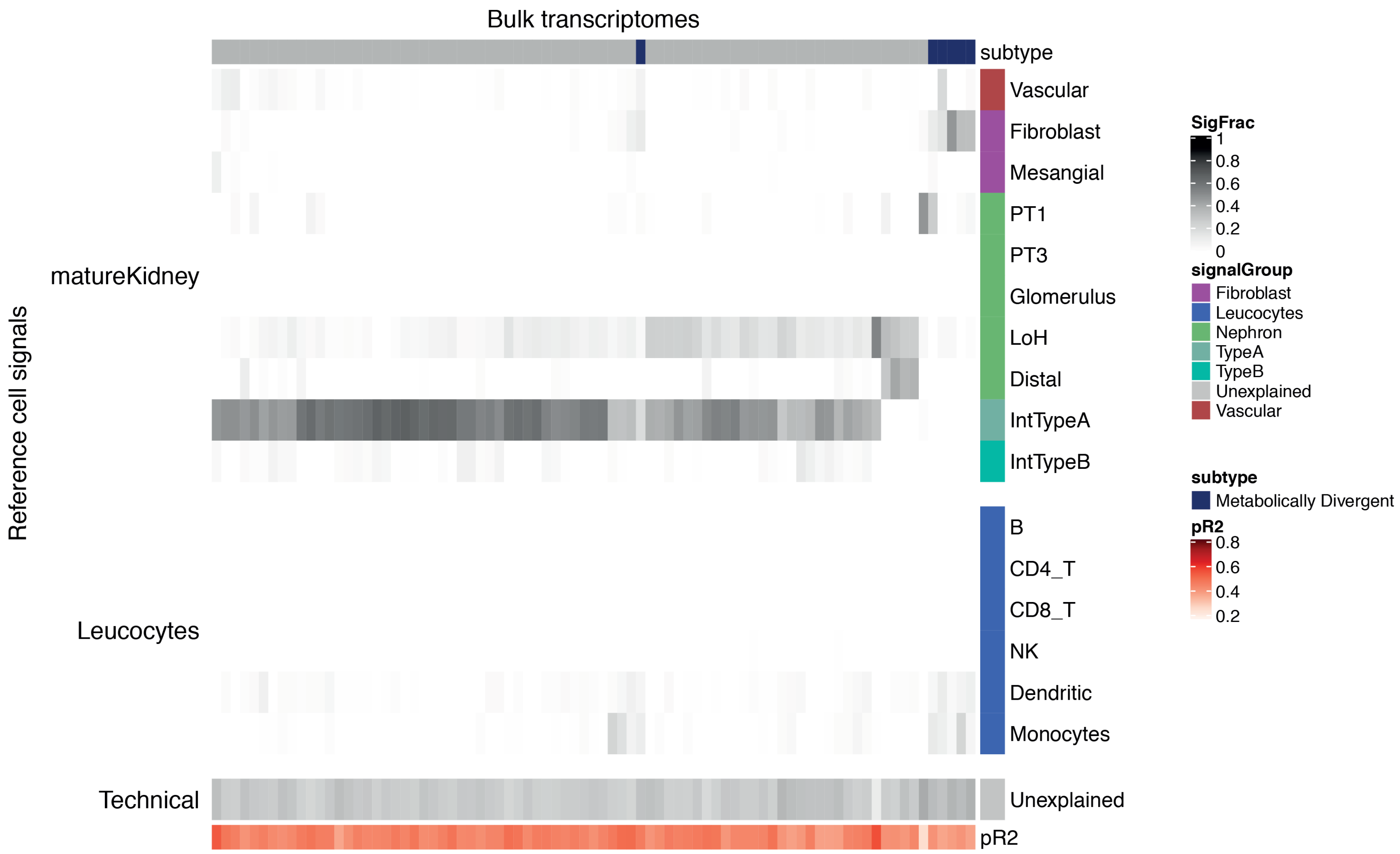

Bulk transcriptomes
subtype
Reference cell signals
matureKidney
Vascular
Fibroblast
Mesangial
PT1
PT3
Glomerulus
LoH
Distal
IntTypeA
IntTypeB
Leucocytes
B
CD4_T
CD8_T
NK
Dendritic
Monocytes
Technical
Unexplained
pR2
SigFrac
1
0.8
0.6
0.4
0.2
0
signalGroup
Fibroblast
Leucocytes
Nephron
TypeA
TypeB
Unexplained
Vascular
subtype
Metabolically Divergent
pR2
0.8
0.6
0.4
0.2

Supplementary Figure 14 – ChRCC fit using mature kidney signals

The same data as in **Fig. 6E**, but presented in heatmap form. Each column represents a sample and each row a collection of reference signal. Subtypes of tumor are indicated by the annotation row at the top of the figure. The shading in each cell represents the relative contribution of that collection of reference signals to explaining the bulk transcriptome of that sample. The pR2 column represents a pseudo-R squared value for each sample, calculated as 1 minus the ratio of the log likelihoods of the full model to a model consisting of only the intercept term. Source data are available as a Source Data file.

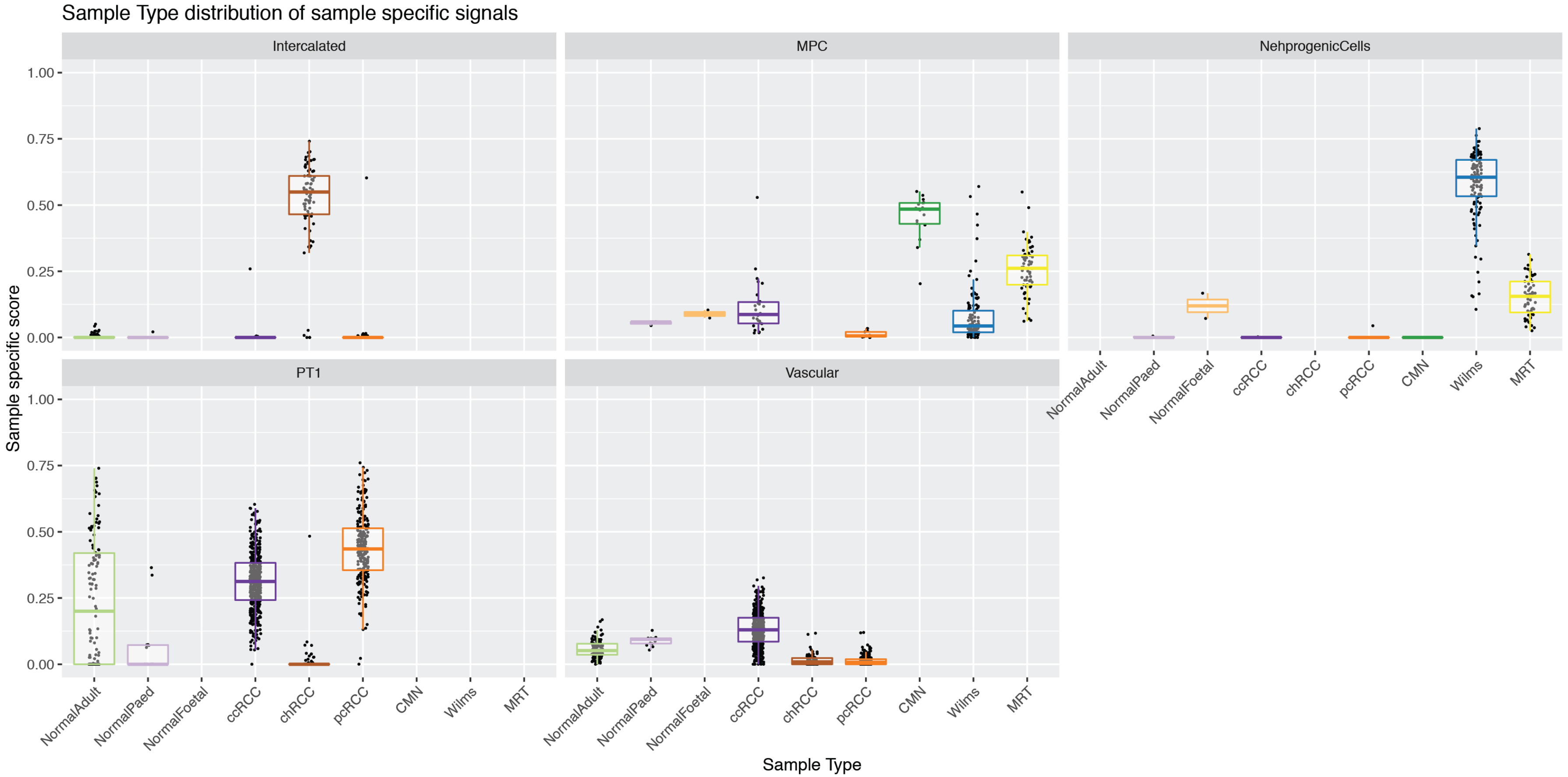

Supplementary Figure 15 – Specificity of signal contributions to tumor types

Each panel shows the distribution of the contribution of different "scores" calculated by aggregating the contribution from various signals, across a range of different tumor and normal samples. Each point represents a sample, with its y-axis value giving its score, with the score type given by the panel. Samples are broken down by sample/tumor type as shown on the x-axis, with each sample type given a different color. Box-plots show the distribution of the scores within each sample type. This is a more detailed version of the data shown in **Fig. 7B**. Boxplots show the distribution median (middle line), 1st and 3rd quartiles (box limits), and 1.5 times the inter-quartile range beyond the box-limits (whiskers). Source data are available as a Source Data file.

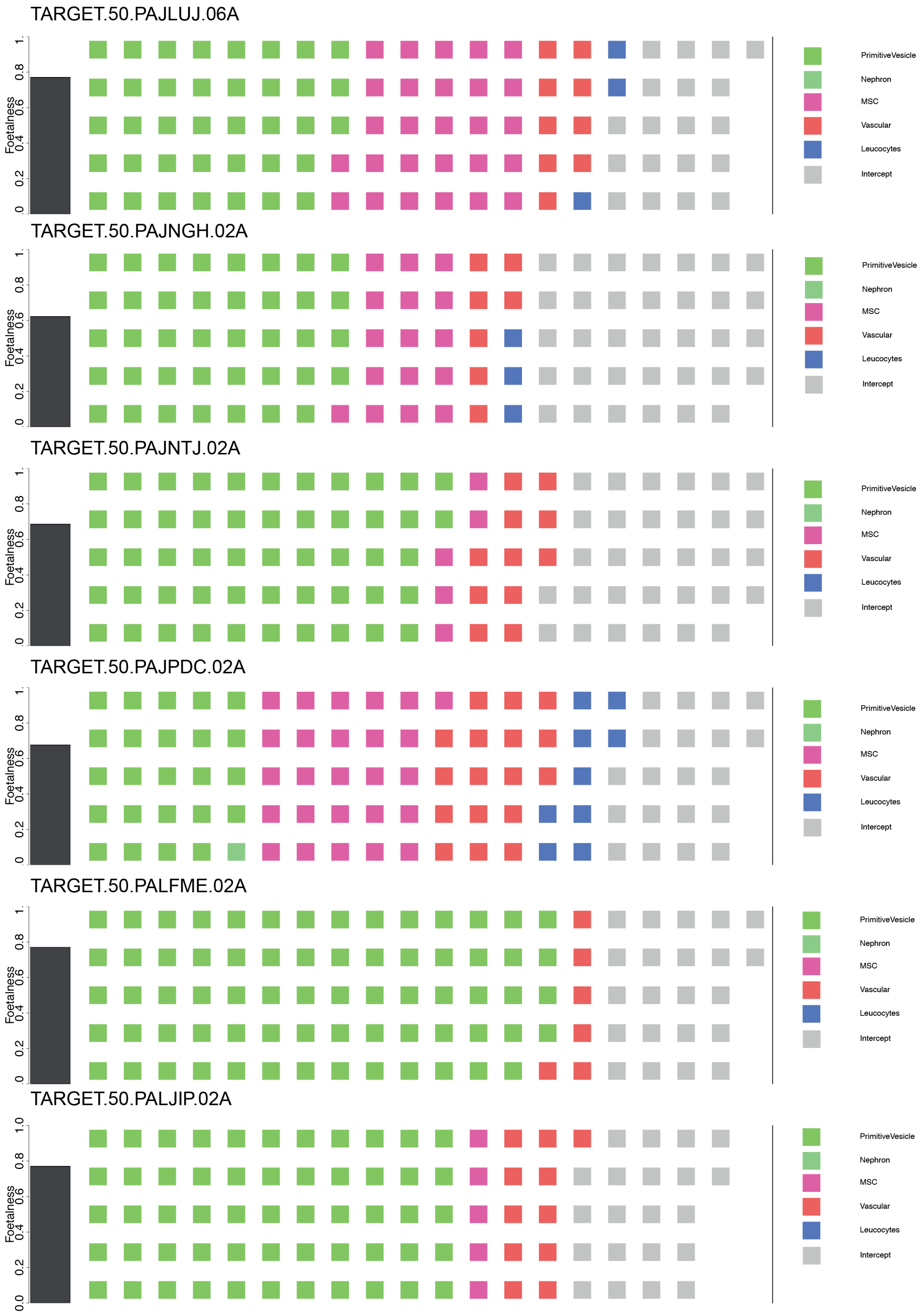
TARGET.50.PAJLUJ.06A
TARGET.50.PAJNGH.02A
TARGET.50.PAJNTJ.02A
TARGET.50.PAJPDC.02A
TARGET.50.PALFME.02A
TARGET.50.PALJIP.02A
Foetalness
0
0.2
0.4
0.6
0.8
1.
0.0
1.0
PrimitiveVesicle
Nephron
MSC
Vascular
Leucocytes
Intercept

Supplementary Figure 16 – Additional validation of tumor type inference
The fetalness score and cell signal contribution for 6 non-primary Wilms tumors calculated as in **Fig. 7D-G**. The contribution from each cell signal to each bulk transcriptome was multiplied by 100 and rounded down to the nearest integer, which then determined the number of squares shown for each sample. The type of signal each square represents is indicated by the legend on the right.
Source data are available as a Source Data file.

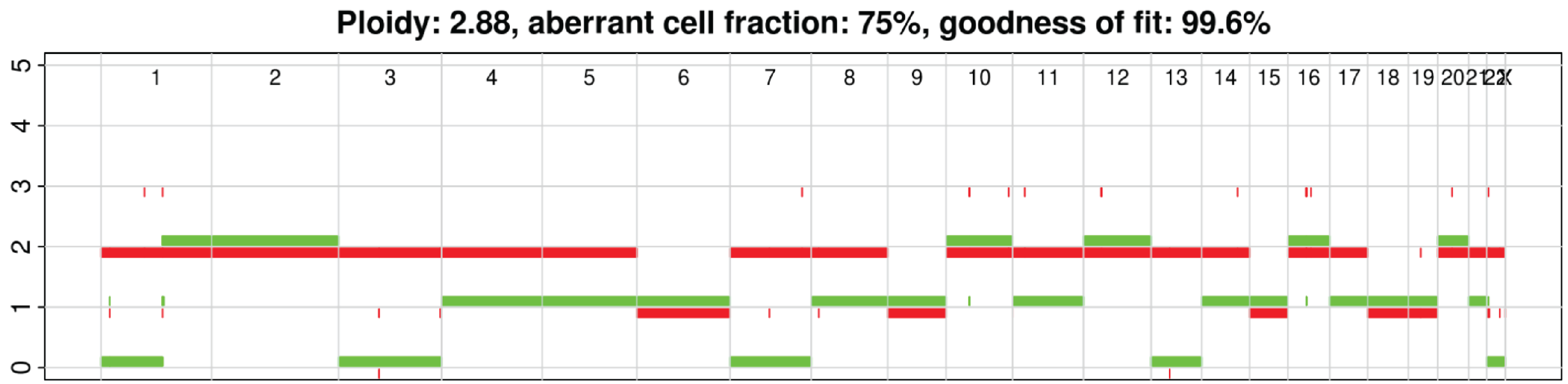

Supplementary Figure 17 – CN profile of unknown childhood renal tumor
Copy number profile of the unknown childhood renal tumor (SangerProject1700_PR40309a). The colored lines represent the major (red) and minor (green) alleles copy number (y-axis) along the chromosomes as shown at the top of the plot.

Colour scheme for cellular signals

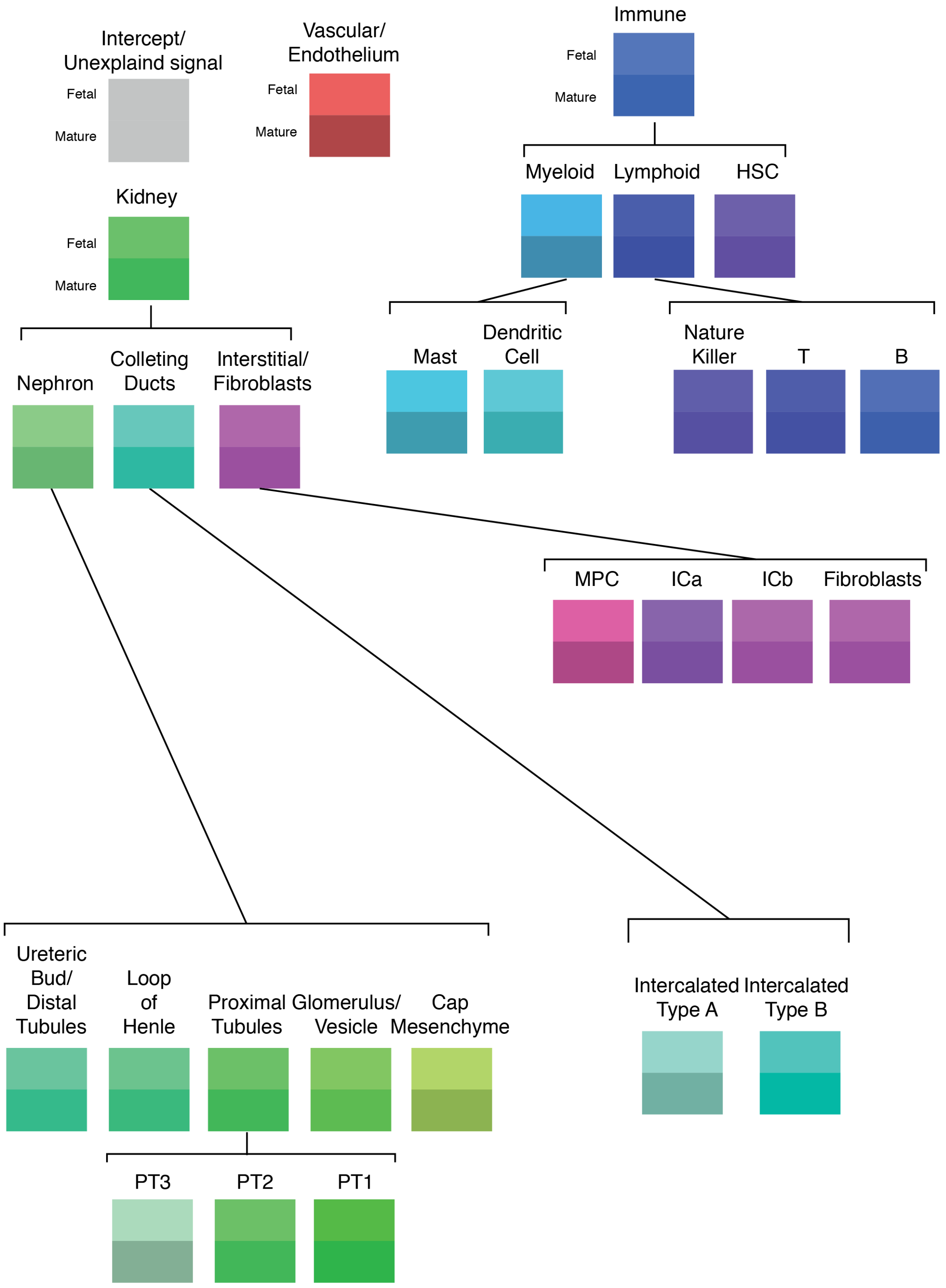

Supplementary Figure 18 – Color scheme for cell types
Overview of the color scheme used throughout the figures in this paper to represent different cell types. Cell types are organized hierarchically, with fetal/mature versions of the same cell type represented by colors of the same hue and saturation but different value. The exception to this rule is the unexplained signal color which is the same in both a fetal and mature context.

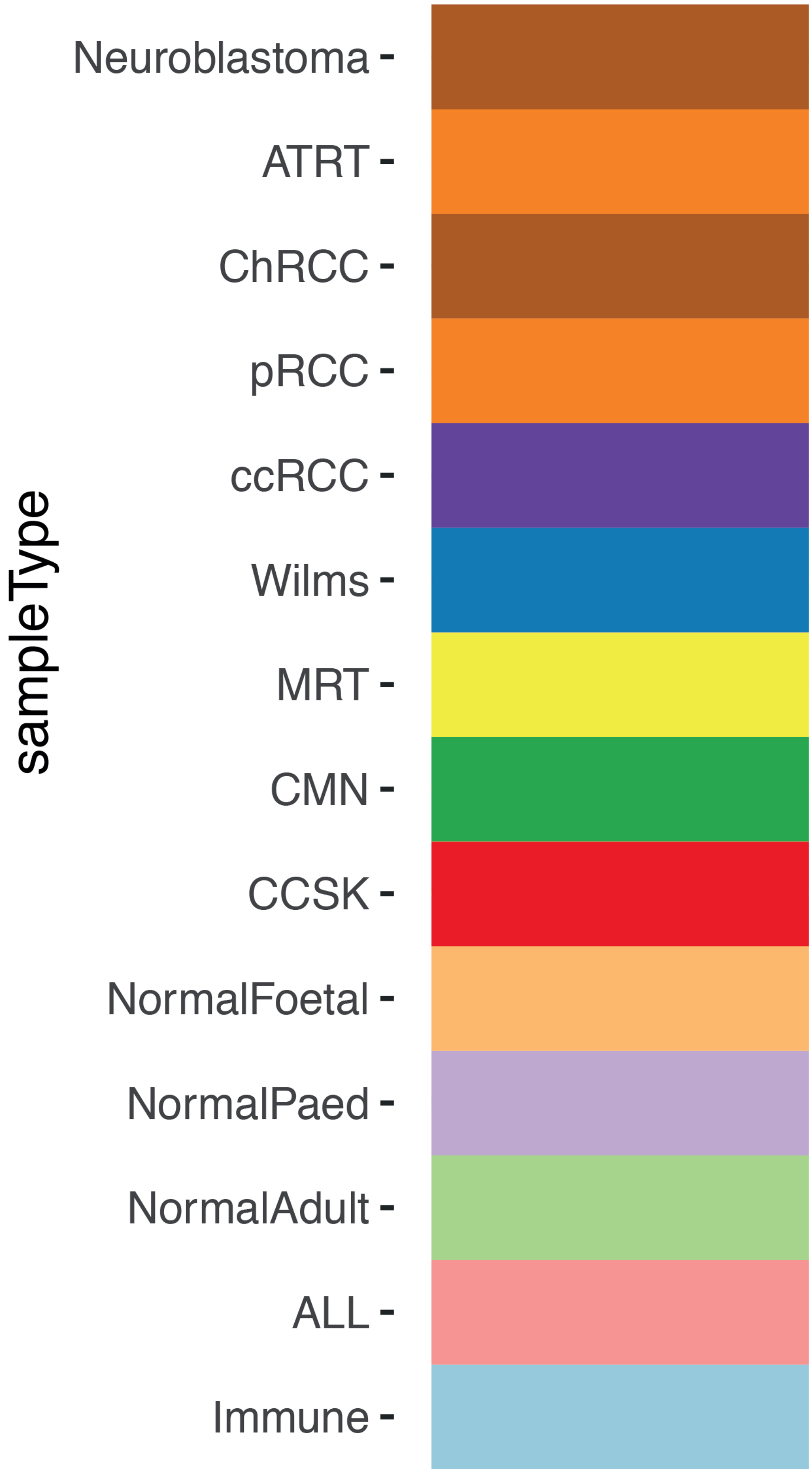

Supplementary Figure 19 – Color scheme for sample types
Overview of the color scheme used throughout the figures in this paper to represent different sample types. Where colors have been duplicated they either never occur in the same context, or it is obvious from the context which color refers to which sample type.

channelLabel	patientLabel	sampleType	disease	age	sort	dataSource	assay
4834STDY7002873	Foetus15	Normal	Normal	12 PCW	CD45+	DOI: 10.1126/science.aat1699	10X 3'
4834STDY7002874	Foetus15	Normal	Normal	12 PCW	CD45-	DOI: 10.1126/science.aat1699	10X 3'
4834STDY7002875	Foetus16	Normal	Normal	8+1 PCW	CD45+	DOI: 10.1126/science.aat1699	10X 3'
4834STDY7002876	Foetus16	Normal	Normal	8+1 PCW	CD45-	DOI: 10.1126/science.aat1699	10X 3'
4834STDY7002881	Foetus17	Normal	Normal	9+1 PCW	None	DOI: 10.1126/science.aat1699	10X 3'
4834STDY7002885	Foetus17	Normal	Normal	9+1 PCW	CD45+	DOI: 10.1126/science.aat1699	10X 3'
4834STDY7002886	Foetus17	Normal	Normal	9+1 PCW	CD45-	DOI: 10.1126/science.aat1699	10X 3'
FCAImmP7462242	Foetus35	Normal	Normal	7+6 PCW	CD45+	DOI: 10.1126/science.aat5031	10X 3'
FCAImmP7462243	Foetus35	Normal	Normal	7+6 PCW	CD45-	DOI: 10.1126/science.aat5031	10X 3'
FCAImmP7528292	Foetus38	Normal	Normal	13+6 PCW	CD45+	DOI: 10.1126/science.aat5031	10X 3'
FCAImmP7528293	Foetus38	Normal	Normal	13+6 PCW	CD45-	DOI: 10.1126/science.aat5031	10X 3'
FCAImmP7555849	Foetus41	Normal	Normal	16+6 PCW	CD45+	DOI: 10.1126/science.aat5031	10X 3'
FCAImmP7555850	Foetus41	Normal	Normal	16+6 PCW	CD45-	DOI: 10.1126/science.aat5031	10X 3'
FCAImmP7579214	Foetus45	Normal	Normal	13+6 PCW	CD45+	DOI: 10.1126/science.aat5031	10X 3'
FCAImmP7579215	Foetus45	Normal	Normal	13+6 PCW	CD45-	DOI: 10.1126/science.aat5031	10X 3'
w09	w09	Normal	Normal	NA	NA	DOI: 10.1371/journal.pbio.3000152	10X 3'
w11	w11	Normal	Normal	NA	NA	DOI: 10.1371/journal.pbio.3000152	10X 3'
w13	w13	Normal	Normal	NA	NA	DOI: 10.1371/journal.pbio.3000152	10X 3'
w16	w16	Normal	Normal	NA	NA	DOI: 10.1371/journal.pbio.3000152	10X 3'
w18	w18	Normal	Normal	NA	NA	DOI: 10.1371/journal.pbio.3000152	10X 3'
4602STDY7685338	PD42187	Tumour	CMN	NA	None	EGAS00001002325	10X 3'
4602STDY7685339	PD42187	Tumour	CMN	NA	None	EGAS00001002325	10X 3'
5640STDY7891141	MRT1	NormalOrganoid	MRT	NA	None	EGAS00001003386	10X 3'
5640STDY7891142	MRT1	TumourOrganoid	MRT	NA	None	EGAS00001003386	10X 3'
NB8350521	PD48777	Tumour	Wilms	NA	None	EGAD00001007572	10X 3'
NB8350522	PD48777	Tumour	Wilms	NA	CD45-	EGAD00001007572	10X 3'

NB8350523	PD48777	Tumour	Wilms	NA	CD45-	EGAD00001007572	10X 3'
NB8368876	PD48777	Tumour	Wilms	NA	None	EGAD00001007572	10X 3'
NB8368877	PD48777	Tumour	Wilms	NA	CD45-	EGAD00001007572	10X 3'
NB8711738	CCSK1	Tumour	CCSK	NA	NA	EGAD00001007572	Nuclei 10X 3'
NB8711739	CCSK1	Tumour	CCSK	NA	NA	EGAD00001007572	Nuclei 10X 3'
NB8711740	CCSK2	Tumour	CCSK	NA	NA	EGAD00001007572	Nuclei 10X 3'
NB8711741	CCSK2	Tumour	CCSK	NA	NA	EGAD00001007572	Nuclei 10X 3'
NB8711742	CCSK3	Tumour	CCSK	NA	NA	EGAD00001007572	Nuclei 10X 3'
TM211	1015T1	Tumour	CCSK	NA	NA	EGAD00001007498	CEL-Seq2
TM212	1015T1	Tumour	CCSK	NA	NA	EGAD00001007498	CEL-Seq2
NB8350518	PD47704	Tumour	MRT	NA	None	EGAD00001007572	10X 3'
NB8350519	PD47704	Tumour	MRT	NA	CD45-	EGAD00001007572	10X 3'
NB8350520	PD47704	Tumour	MRT	NA	CD45-	EGAD00001007572	10X 3'
NB8368300	PD47704	Tumour	MRT	NA	None	EGAD00001007572	10X 3'
NB8368301	PD47704	Tumour	MRT	NA	CD45-	EGAD00001007572	10X 3'
NB8711736	MRT3	Tumour	MRT	NA	NA	EGAD00001007572	Nuclei 10X 3'
NB8711737	MRT3	Tumour	MRT	NA	NA	EGAD00001007572	Nuclei 10X 3'

Supplementary Table 1 – Single cell manifest
A table giving a description of each single cell experiment included in this study. In the age column, PCW stands for post conception weeks. For each row, the raw count data is available in **Supplementary Data 3** in a folder with name given by the "channelLabel" column.

coefficient	Estimate	Std. Error	t value	Pr(>|t|)	qVal
(Intercept)	0.043420795	0.007086455	6.127293879	1.34E-09	1.05E-08
splitccRCC	0.097857036	0.007328719	13.35254347	3.22E-37	1.16E-35
splitpRCC CIMP	0.142529785	0.023561658	6.049225545	2.14E-09	1.28E-08
splitpRCC T1	0.108900758	0.008940814	12.1801836	1.09E-31	1.97E-30
splitpRCC T2	0.0410931	0.010917653	3.763913481	0.00017818	0.000916353
splitpRCC Unc	0.085075024	0.0139183	6.112458078	1.46E-09	1.05E-08
splitchRCC	-0.016045051	0.010752786	-1.492176164	0.13600536	0.30601206
splitchRCC MD	0.271093348	0.041082107	6.598818053	7.09E-11	8.51E-10
hitTypeTP53singleHit	0.021047117	0.011677218	1.802408447	0.0718175	0.215452499
hitTypeTP53doubleHit	0.000687359	0.021635183	0.031770449	0.974662196	0.974662196
hitTypeBAP1singleHit	-0.013486051	0.008870115	-1.520391994	0.128765671	0.30601206
hitTypeBAP1doubleHit	-0.069745425	0.049447887	-1.410483414	0.158744444	0.317488888
hitTypeCDKN2AsingleHit	0.024653553	0.025087408	0.982706237	0.32601761	0.510288433
hitTypeVHLsingleHit	0.006883926	0.006049754	1.137885346	0.255472876	0.459851177
hitTypeVHLdoubleHit	-0.007308534	0.019379223	-0.377132486	0.706164553	0.85176515
hitTypePBRM1singleHit	0.002144084	0.006294524	0.340626871	0.733464435	0.85176515
hitTypePBRM1doubleHit	-0.006196931	0.029184362	-0.212337396	0.831892171	0.907518732
hitTypeSETD2singleHit	0.016482962	0.008195675	2.011178013	0.044606071	0.145983506
hitTypeSETD2doubleHit	-0.008720474	0.025086187	-0.347620563	0.728206894	0.85176515
hitTypeKDM5CsingleHit	-0.015657612	0.011737054	-1.334032499	0.182532552	0.345851151
hitTypeKDM5CdoubleHit	0.103279661	0.050626133	2.040046403	0.04163883	0.145983506
hitTypePTENsingleHit	0.009161375	0.011702299	0.782869655	0.43391075	0.650866126
hitTypePTENdoubleHit	0.136496255	0.040337911	3.38382063	0.000745889	0.003356502
hitTypeMTORsingleHit	-0.007429789	0.011244737	-0.66073476	0.508952457	0.732891538
hitTypeMTORdoubleHit	0.023895949	0.049156597	0.486118856	0.627001896	0.806145295
hitTypePIK3CAsingleHit	-0.017528165	0.016367274	-1.070927538	0.284490719	0.487698376
hitTypePIK3CAdoubleHit	-0.119272346	0.071174849	-1.675765351	0.094133438	0.260677213
hitTypeMETsingleHit	0.013717814	0.013809371	0.993369928	0.320798196	0.510288433
hitTypeMETdoubleHit	0.010675768	0.049227288	0.216866867	0.828361523	0.907518732
hitTypeFAT1singleHit	0.000809979	0.012684051	0.063858047	0.949097517	0.974662196
hitTypeNF2singleHit	0.036993869	0.017774285	2.081313973	0.037688938	0.145983506
hitTypeKDM6AsingleHit	-0.030125498	0.019992242	-1.506859435	0.132199554	0.30601206
hitTypeKDM6AdoubleHit	-0.003423503	0.050729383	-0.067485599	0.946210199	0.974662196
hitTypeSMARCB1singleHit	-0.028075701	0.019652678	-1.428594183	0.153469791	0.317488888
hitTypeNFE2L2singleHit	-0.009935681	0.018581713	-0.534702086	0.592988685	0.790651579
hitTypeSTAG2singleHit	-0.009271449	0.016090918	-0.576191427	0.564630661	0.7817963

Supplementary Table 2 – Linear model of immaturity by genotype

Generated using “summary(fit)” in R, where fit is a linear model to predict the immaturity score of RCCs using sample genotype as covariates. Covariates are coded such that the intercept term corresponds to the wild type of non-tumor biopsy.

coefficient	Estimate	Std. Error	t value	Pr(>|t|)	qVal
(Intercept)	0.163510869	0.018861217	8.669158019	6.91E-17	1.11E-15
mRNA1	0.023556367	0.010863869	2.168322037	0.030629906	0.08167975
mRNA2	0.001214761	0.011694473	0.103874825	0.917312529	0.993826308
mRNA3	0.061739964	0.011961718	5.161463027	3.61E-07	2.89E-06
mRNA4	-0.028420201	0.01216071	-2.3370511	0.019851151	0.068526977
miRNA1	0.033287926	0.01161994	2.864724376	0.004358656	0.023246165
miRNA2	-0.008154287	0.011496292	-0.709297152	0.478488096	0.695982686
miRNA3	-0.010115854	0.010740307	-0.941858989	0.346743598	0.554789757
miRNA4	0.015538685	0.011823078	1.314267285	0.18939056	0.336694329
gradeG2	-0.025578429	0.019110589	-1.33844272	0.181392447	0.336694329
gradeG3	-0.044934636	0.019467088	-2.308236131	0.02141468	0.068526977
gradeG4	-0.042089242	0.021361856	-1.970298941	0.049384495	0.112878847
gradeGX	-0.001147628	0.066465078	-0.017266634	0.986231154	0.993826308
stageStage II	0.000917833	0.010061156	0.091225427	0.927351929	0.993826308
stageStage III	-9.37E-05	0.007659532	-0.012228755	0.990248243	0.993826308
stageStage IV	7.37E-05	0.009513506	0.007741727	0.993826308	0.993826308

Supplementary Table 3 – Linear model of immaturity by other features
Generated using “summary(fit)” in R, where fit is a linear model to predict the immaturity score of ccRCCs using sample genotype as covariates. Covariates are coded such that the intercept term corresponds to unknown mRNA group, unknown miRNA group, grade G1, and stage I.

tType	nCells	nMast	mastFrac
pRCC2	1215037	8598	0.007076328
ccRCC	645654	48862	0.075678304
pRCC1	686715	57156	0.083231035

Supplementary Table 4 – Mast cell fraction for RCCs
Quantification of mast cell prevalence from smFISH of different tumors. In the column "tType" pRCC1/2 represents papillary cell renal cell carcinoma type 1/2.

ID	Disease	Assay
PD42187	CMN	SingleCellTranscriptomics
MRT1	MRT	SingleCellTranscriptomics
PD48777	Wilms	SingleCellTranscriptomics
CCSK1	CCSK	SingleCellTranscriptomics
CCSK2	CCSK	SingleCellTranscriptomics
CCSK3	CCSK	SingleCellTranscriptomics
1015T1	CCSK	SingleCellTranscriptomics
PD47704	MRT	SingleCellTranscriptomics
MRT3	MRT	SingleCellTranscriptomics
PR36165	Wilms	BulkTranscriptomics
PR37104	ccRCC	BulkTranscriptomics
PR37228	ccRCC	BulkTranscriptomics
PR37272	Wilms	BulkTranscriptomics
PR37276	Wilms	BulkTranscriptomics
PR40710	Wilms	BulkTranscriptomics
PR40712	Wilms	BulkTranscriptomics
PR40713	Wilms	BulkTranscriptomics
PR40715	Wilms	BulkTranscriptomics
PR40716	Wilms	BulkTranscriptomics
PR40717	Wilms	BulkTranscriptomics
PR40718	Wilms	BulkTranscriptomics
PR40719	Wilms	BulkTranscriptomics
PR40720	Wilms	BulkTranscriptomics
PR40722	Wilms	BulkTranscriptomics
PR40726	Wilms	BulkTranscriptomics
PR40727	Wilms	BulkTranscriptomics
PR40728	Wilms	BulkTranscriptomics
PR40729	Wilms	BulkTranscriptomics
PR37201	CMN	BulkTranscriptomics
PR37205	CMN	BulkTranscriptomics
PR37217	CMN	BulkTranscriptomics
PR37220	CMN	BulkTranscriptomics
PR37223	CMN	BulkTranscriptomics
PR37199	CMN	BulkTranscriptomics
PR37200	CMN	BulkTranscriptomics
PR37204	CMN	BulkTranscriptomics
PR37208	CMN	BulkTranscriptomics
PR37212	CMN	BulkTranscriptomics
PR37213	CMN	BulkTranscriptomics

PR37214	CMN	BulkTranscriptomics
PR37216	CMN	BulkTranscriptomics
PR37219	CMN	BulkTranscriptomics
PR37221	CMN	BulkTranscriptomics
PR37222	CMN	BulkTranscriptomics
PR37224	CMN	BulkTranscriptomics
PR37210	CMN	BulkTranscriptomics
PR37225	CMN	BulkTranscriptomics
PR37202	CMN	BulkTranscriptomics
PR40309	CrypticRenalTumor	BulkTranscriptomics

Supplementary Table 5 – Patient samples
A list of all the patient samples used in this study, giving disease type and assay performed.

Supplementary References

1. Hochane, M. *et al.* Single-cell transcriptomics reveals gene expression dynamics of human fetal kidney development. *PLoS Biol.* **17**, (2019).
