# Supplementary material for: Single cell derived mRNA signals across human kidney tumors
Source: Nat Commun. 2021 Jun 23;12:3896. doi: 10.1038/s41467-021-23949-5 (PMC8222373; doi:10.1038/s41467-021-23949-5)
Supplement: Supplementary file 3 — Descriptions of Additional Supplementary Files [file 41467_2021_23949_MOESM3_ESM.pdf]

Descriptions of Additional Supplementary Files

Supplementary data 1
Description: An excel spreadsheet containing a table giving a description of each bulk RNA transcriptome used in this study as well as the path to the file containing the table of counts for that file (which can be extracted from Supplementary Data 2). Also include are Supplementary Tables 1-5 in a more convenient format.

Supplementary data 2
Description: Bulk transcriptomes. Text files giving fragment counts and gene lengths for every sample in this study, with each sample's count table stored in a separate file. Metadata relating to each sample is in Supplementary Dataset 1.

Supplementary data 3
Description: Single cell transcriptomes. Tables of counts and QC metrics for every single cell dataset generated in this study. The count tables are stored in h5 format, stored one folder per channel. Metadata relating to each sample is in Supplementary Table 2.

Supplementary Software
Description: Collection of scripts used to perform the analysis and generate this manuscript. These scripts are provided to give additional details as to how we implemented the analyses described in the Methods section.
