# Supplementary material for: Single cell derived mRNA signals across human kidney tumors
Source: Nat Commun. 2021 Jun 23;12:3896. doi: 10.1038/s41467-021-23949-5 (PMC8222373; doi:10.1038/s41467-021-23949-5)
Supplement: Supplementary file 8 — Reporting Summary [file 41467_2021_23949_MOESM8_ESM.pdf]

nature research

Corresponding author(s): Matthew Young
Jarno Drost
Karin Straathof
Sam Behjati

Last updated by author(s): Mar 8, 2021

Reporting Summary

Nature Research wishes to improve the reproducibility of the work that we publish. This form provides structure for consistency and transparency in reporting. For further information on Nature Research policies, see our Editorial Policies and the Editorial Policy Checklist.

Statistics

For all statistical analyses, confirm that the following items are present in the figure legend, table legend, main text, or Methods section.

n/a	Confirmed	
☐	☒	The exact sample size (*n*) for each experimental group/condition, given as a discrete number and unit of measurement
☐	☒	A statement on whether measurements were taken from distinct samples or whether the same sample was measured repeatedly
☐	☒	The statistical test(s) used AND whether they are one- or two-sided<br>*Only common tests should be described solely by name; describe more complex techniques in the Methods section.*
☐	☒	A description of all covariates tested
☐	☒	A description of any assumptions or corrections, such as tests of normality and adjustment for multiple comparisons
☐	☒	A full description of the statistical parameters including central tendency (e.g. means) or other basic estimates (e.g. regression coefficient) AND variation (e.g. standard deviation) or associated estimates of uncertainty (e.g. confidence intervals)
☐	☒	For null hypothesis testing, the test statistic (e.g. *F*, *t*, *r*) with confidence intervals, effect sizes, degrees of freedom and *P* value noted<br>*Give P values as exact values whenever suitable.*
☒	☐	For Bayesian analysis, information on the choice of priors and Markov chain Monte Carlo settings
☒	☐	For hierarchical and complex designs, identification of the appropriate level for tests and full reporting of outcomes
☒	☐	Estimates of effect sizes (e.g. Cohen's *d*, Pearson's *r*), indicating how they were calculated

Our web collection on statistics for biologists contains articles on many of the points above.

Software and code

Policy information about availability of computer code

Data collection	No software was used for data collection.
Data analysis	This is described in detail in the supplementary material and documented in the provided code. The analysis was performed using the R statistical computing language (v3) and cellular signal analysis was run using the tensorflow (v2) framework in python (v3). We also used CaveMan v1.11.2, ASCATngs v4.0.1, Battenberg v2.2.5, cellranger v2.0.2 and v3.0.2, and Seurat v3.1.4.

For manuscripts utilizing custom algorithms or software that are central to the research but not yet described in published literature, software must be made available to editors and reviewers. We strongly encourage code deposition in a community repository (e.g. GitHub). See the Nature Research guidelines for submitting code & software for further information.

Data

Policy information about availability of data

All manuscripts must include a data availability statement. This statement should provide the following information, where applicable:

- Accession codes, unique identifiers, or web links for publicly available datasets
- A list of figures that have associated raw data
- A description of any restrictions on data availability

Mapped count data (i.e tables of counts) are available as Data S1 (bulk transcriptomes) and Data S2 (single cell transcriptomes). Sample metadata, and EGA accession numbers where raw nucleotide sequences can be accessed or references to the originating dataset are listed for each unit of data in Table S1 (bulk RNA seq) and Table S2 (single cell RNA seq). Refer to these tables for which sample is available at which accession number/link. The key accession numbers are: EGAS00001002715, EGAS00001002325, EGAS00001003386, EGAS00001002534, EGAS00001002487. The source data that were used to generate all figures in this manuscript are made available in Data S3.

Field-specific reporting

Please select the one below that is the best fit for your research. If you are not sure, read the appropriate sections before making your selection.

- [x] Life sciences
- [ ] Behavioural & social sciences
- [ ] Ecological, evolutionary & environmental sciences

For a reference copy of the document with all sections, see nature.com/documents/nr-reporting-summary-flat.pdf

Life sciences study design

All studies must disclose on these points even when the disclosure is negative.

Sample size	Sample sizes were primarily determined by the availability of data, either through publicly available databases such as TCGA or TARGET, or through our own sample collection efforts.
Data exclusions	No data exclusions, except for standard QC filters (see supplementary methods).
Replication	Replication of experiments was often not possible due to the rare nature of the kidney tumours studied. We obtained 3 replicates for the CCSK single cell data and 3 replicates for MRT single cell data.
Randomization	The only "experimental group" in our study was the type of tumour a patient had, which it clearly not possible to randomise or blind to.
Blinding	The only "experimental group" in our study was the type of tumour a patient had, which it clearly not possible to randomise or blind to.

Reporting for specific materials, systems and methods

We require information from authors about some types of materials, experimental systems and methods used in many studies. Here, indicate whether each material, system or method listed is relevant to your study. If you are not sure if a list item applies to your research, read the appropriate section before selecting a response.

Materials & experimental systems

n/a	Involved in the study	
☐	☒	Antibodies
☒	☐	Eukaryotic cell lines
☒	☐	Palaeontology and archaeology
☒	☐	Animals and other organisms
☐	☒	Human research participants
☒	☐	Clinical data
☒	☐	Dual use research of concern

Methods

n/a	Involved in the study	
☒	☐	ChIP-seq
☒	☐	Flow cytometry
☒	☐	MRI-based neuroimaging

Antibodies

Antibodies used	INI-1 (BD Transduction Laboratories, 612111, 1:400) or TWIST (Abcam, ab50581, 1:500)
Validation	All antibodies used in this study were validated by the manufacturers. Additionally, on the manufacturer's antibody web page, references to other studies that made use of the antibody are provided.<br>https://www.bdbiosciences.com/us/reagents/research/antibodies-buffers/cell-biology-reagents/cell-biology-antibodies/purified-mouse-anti-baf47-25baf47/p/612111 [bdbiosciences.com]<br>https://www.abcam.com/twist-antibody-ab50581.html [abcam.com]

Human research participants

Policy information about studies involving human research participants

Population characteristics	These population covariates are provided in the supplementary data and are too extensive to list here. See TCGA and TARGET for more details.
Recruitment	Patients were recruited through the authors clinical practice or the HDBR (http://ww.hdbr.org).
Ethics oversight	All human material was obtained through ethically approved studies with the following references: NHS National Research Ethics Service reference 03/018 (DIAMOND study; adult kidney tissues); NHS National Research Ethics Service reference 16/EE/0394 (pediatric tissues); NHS National Research Ethics Service reference 96/085 (fetal tissues). Organoids were generated from human tissue as approved by the medical ethics committee of the Erasmus Medical Center (Rotterdam, the Netherlands). Additional fetal tissue was provided by the Joint MRC / Wellcome Trust-funded (grant # 099175/Z/12/Z) Human Developmental BiologyResource (HBDR, http://www.hdbr.org; (10)), with appropriate maternal written consent and approval from the Newcastle and North Tyneside NHS Health Authority Joint Ethics Committee. HDBR is regulated by the UK Human Tissue Authority (HTA;www.hta.gov.uk) and operates in accordance with the relevant HTA Codes of Practice.

Note that full information on the approval of the study protocol must also be provided in the manuscript.
