# Supplementary material for: Single cell derived mRNA signals across human kidney tumors
Source: Nat Commun. 2021 Jun 23;12:3896. doi: 10.1038/s41467-021-23949-5 (PMC8222373; doi:10.1038/s41467-021-23949-5)
Supplement: Supplementary file 6 — Supplementary Data 3 [file 41467_2021_23949_MOESM6_ESM.zip › SupplementaryData3/FCAImmP7462242/web_summary.html]

FCAImmP7462242 — Cell Ranger
 


Cell Ranger · FCAImmP7462242 ·

{{ alerts.length}}

SUMMARY
    
ANALYSIS

The analysis detected some serious issues.  
 Details »

| Alert | | Value | Detail |
| --- | --- | --- | --- |
|  | **{{ alert.title }}** | {{ alert.value }} | {{ alert.message }} |

# Estimated Number of Cells

1,970

## Mean Reads per Cell

191,432

## Median Genes per Cell

1,521

Number of Reads
:   Total number of single-end reads that were assigned to this library in demultiplexing.

Valid Barcodes
:   Fraction of reads with barcodes that match the whitelist after barcode correction.

Reads Mapped Confidently to Transcriptome
:   Fraction of reads that mapped to a unique gene in the transcriptome with a high mapping quality score as reported by the aligner. At least 50% of the read must overlap with an exon and the read must be consistent with annotated splice junctions.

Reads Mapped Confidently to Exonic Regions
:   Fraction of reads that mapped to the exonic regions of the genome with a high mapping quality score as reported by the aligner.

Reads Mapped Confidently to Intronic Regions
:   Fraction of reads that mapped to the intronic regions of the genome with a high mapping quality score as reported by the aligner.

Reads Mapped Confidently to Intergenic Regions
:   Fraction of reads that mapped to the intergenic regions of the genome with a high mapping quality score as reported by the aligner.

Reads Mapped Antisense to Gene
:   Fraction of reads confidently mapped to the transcriptome, but on the opposite strand of their annotated gene.

Sequencing Saturation
:   The fraction of reads originating from an already-observed UMI. This is a function of library complexity and sequencing depth. More specifically, this is the fraction of confidently mapped, valid cell-barcode, valid UMI reads that had a non-unique (cell-barcode, UMI, gene). This metric was called "cDNA PCR Duplication" in versions of Cell Ranger prior to 1.2.

Q30 Bases in Barcode
:   Fraction of cell barcode bases with Q-score >= 30, excluding very low quality/no-call (Q <= 2) bases from the denominator.

Q30 Bases in RNA Read
:   Fraction of RNA read bases with Q-score >= 30, excluding very low quality/no-call (Q <= 2) bases from the denominator. This is Read 1 for the Single Cell 3' v1 chemistry and Read 2 for the Single Cell 3' v2 chemistry.

Q30 Bases in Sample Index
:   Fraction of sample index bases with Q-score >= 30, excluding very low quality/no-call (Q <= 2) bases from the denominator.

Q30 Bases in UMI
:   Fraction of UMI bases with Q-score >= 30, excluding very low quality/no-call (Q <= 2) bases from the denominator.

# Sequencing

|  |  |
| --- | --- |
| Number of Reads | 377,122,282 |
| Valid Barcodes | 98.3% |
| Reads Mapped Confidently to Transcriptome | 63.9% |
| Reads Mapped Confidently to Exonic Regions | 67.5% |
| Reads Mapped Confidently to Intronic Regions | 17.0% |
| Reads Mapped Confidently to Intergenic Regions | 3.3% |
| Reads Mapped Antisense to Gene | 4.1% |
| Sequencing Saturation | 95.2% |
| Q30 Bases in Barcode | 97.4% |
| Q30 Bases in RNA Read | 74.1% |
| Q30 Bases in Sample Index | 95.2% |
| Q30 Bases in UMI | 97.8% |

Estimated Number of Cells
:   The total number of barcodes associated with cell-containing partitions, estimated from the barcode count distribution.

Fraction Reads in Cells
:   The fraction of valid-barcode, confidently-mapped-to-transcriptome reads with cell-associated barcodes.

Mean Reads per Cell
:   The total number of sequenced reads divided by the number of barcodes associated with cell-containing partitions.

Median Genes per Cell
:   The median number of genes detected per cell-associated barcode. Detection is defined as the presence of at least 1 UMI count.

Total Genes Detected
:   The number of genes with at least one UMI count in any cell.

Median UMI Counts per Cell
:   The median number of UMI counts per cell-associated barcode.

# Cells

|  |  |
| --- | --- |
| Estimated Number of Cells | 1,970 |
| Fraction Reads in Cells | 95.6% |
| Mean Reads per Cell | 191,432 |
| Median Genes per Cell | 1,521 |
| Total Genes Detected | 20,891 |
| Median UMI Counts per Cell | 4,799 |

# Sample

|  |  |
| --- | --- |
| Name | FCAImmP7462242 |
| Description |  |
| Transcriptome | GRCh38 |
| Chemistry | Single Cell 3' v2 |
| Cell Ranger Version | 2.0.2 |

{{ filter.selected }}   

- {{ value }}

#### {{ title }}:

{{ chart.description }}

## {{ chart.title }}

{{ chart.description }}

## {{ chart.title }}

{{ chart.description }}

## {{ chart.title }}

|  |  |  |  |  |
| --- | --- | --- | --- | --- |
|  |  | Cluster {{ $index/2 }} | |  |
| {{ col.label }} |  |  |  |  |
| {{ cell }} | {{ cell.f }} |  |  |  |

{{ chart.description }}

## {{ chart.title }}

{{ chart.description }}

## {{ chart.title }}
